# *In Vivo* Efficacy and Toxicity of Curcumin Nanoparticles in Breast Cancer Treatment: A Systematic Review

**DOI:** 10.3389/fonc.2021.612903

**Published:** 2021-03-09

**Authors:** Alicia S. Ombredane, Vitória R. P. Silva, Laise R. Andrade, Willie O. Pinheiro, Mayara Simonelly, Jaqueline V. Oliveira, Andréia C. Pinheiro, Gabriel F. Gonçalves, Gisela J. Felice, Mônica P. Garcia, Patrícia M. Campos, Glécia V. S. Luz, Graziella A. Joanitti

**Affiliations:** ^1^ Laboratory of Bioactive Compounds and Nanobiotechnology (LBCNano), University of Brasilia, Brasilia, Brazil; ^2^ Post-Graduation Program in Nanoscience and Nanobiotechnology, Institute of Biological Sciences, University of Brasilia, Brasilia, Brazil; ^3^ Department of Genetics & Morphology, Institute of Biological Sciences, University of Brasilia, Brasilia, Brazil; ^4^ Post-Graduation Program in Sciences and Technologies in Health, Faculty of Ceilandia, University of Brasilia, Brasilia, Brazil; ^5^ Department of Immunology, Institute of Biomedical Sciences, University of Sao Paulo, Sao Paulo, Brazil; ^6^ Pharmaceutical Sciences Department, State University of Ponta Grossa, Parana, Brazil; ^7^ Post-Graduate Program in Biomedical Engineering-PPGEB, Faculty of Gama-FGA, University of Brasilia, Brasilia, Brazil; ^8^ Health Technology Assessment Center-NATS/UnB, University of Brasília, Brasilia, Brazil

**Keywords:** breast cancer, nanoparticle, curcumin, apoptosis, antitumor, toxicity, *in vivo*, systematic review

## Abstract

Breast cancer is one of the most prevalent types of malignant tumors in the world, resulting in a high incidence of death. The development of new molecules and technologies aiming to apply more effective and safer therapy strategies has been intensively explored to overcome this situation. The association of nanoparticles with known antitumor compounds (including plant-derived molecules such as curcumin) has been considered an effective approach to enhance tumor growth suppression and reduce adverse effects. Therefore, the objective of this systematic review was to summarize published data regarding evaluations about efficacy and toxicity of curcumin nanoparticles (Cur-NPs) in *in vivo* models of breast cancer. The search was carried out in the databases: CINAHL, Cochrane, LILACS, Embase, FSTA, MEDLINE, ProQuest, BSV regional portal, PubMed, ScienceDirect, Scopus, and Web of Science. Studies that evaluated tumor growth in *in vivo* models of breast cancer and showed outcomes related to Cur-NP treatment (without association with other antitumor molecules) were included. Of the 528 initially gathered studies, 26 met the inclusion criteria. These studies showed that a wide variety of NP platforms have been used to deliver curcumin (*e.g.*, micelles, polymeric, lipid-based, metallic). Attachment of poly(ethylene glycol) chains (PEG) and active targeting moieties were also evaluated. Cur-NPs significantly reduced tumor volume/weight, inhibited cancer cell proliferation, and increased tumor apoptosis and necrosis. Decreases in cancer stem cell population and angiogenesis were also reported. All the studies that evaluated toxicity considered Cur-NP treatment to be safe regarding hematological/biochemical markers, damage to major organs, and/or weight loss. These effects were observed in different *in vivo* models of breast cancer (*e.g.*, estrogen receptor-positive, triple-negative, chemically induced) showing better outcomes when compared to treatments with free curcumin or negative controls. This systematic review supports the proposal that Cur-NP is an effective and safe therapeutic approach in *in vivo* models of breast cancer, reinforcing the currently available evidence that it should be further analyzed in clinical trials for breast cancer treatments.

**Graphical Abstract:**
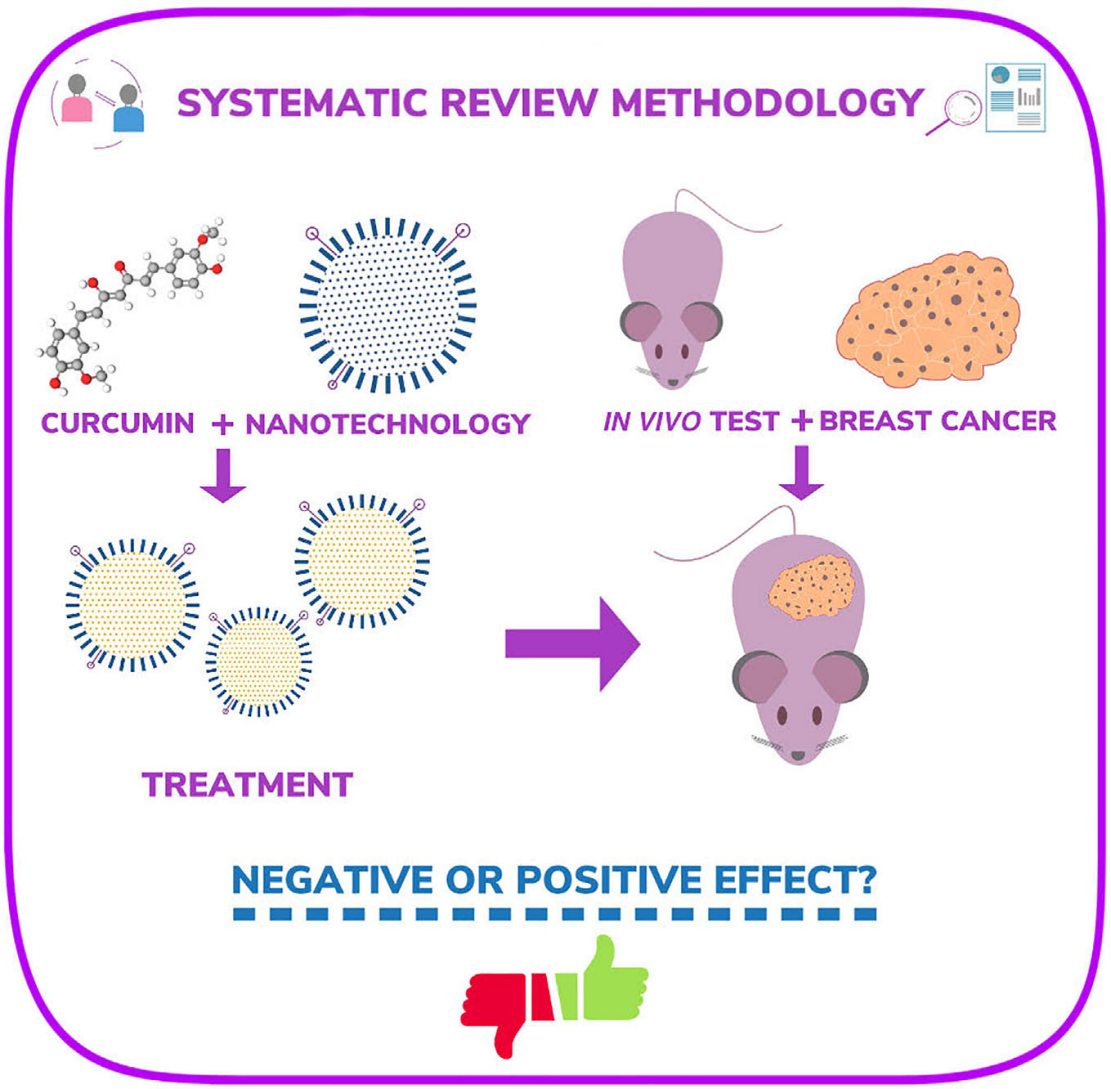


## Introduction

Among women, breast cancer is the most prevalent cancer worldwide, with 2.1 million cases reported in 2018 (with an annual increase of 3.1%) and more than 620,000 deaths per year (1, 2). Breast cancer is divided into five major intrinsic molecular subtypes: luminal A-like (60–70%), luminal B-like human epidermal growth factor receptor-type 2 negative (HER2–) (10–20%), HER2-enriched (non-luminal) and luminal B-like HER2+ (13–15%), and triple-negative (10–15%) (1). Early diagnosis of breast cancer raises the chances of total recuperation of patients, and the treatment concept is decided based on several criteria such as subtype and grade (3). Chemotherapy and endocrine therapy are typical systemic therapies in non-metastatic breast cancer. They can be associated with local therapy like surgery and radiation (3). In most cases, metastatic breast cancer remains incurable and therapy aims to prolong life and alleviate symptoms (3). However, these conventional treatments present some limitations, like resistance to chemotherapy or endocrine therapy, and some adverse effects (4). Thus, alternative treatments must be investigated to improve the recovery of breast cancer patients, reduce adverse effects, and circumvent therapy resistance.

Natural products are considered as promising alternatives for the development of new antitumor drugs (5, 6). Curcumin, or diferuloylmethane, is a yellow pigment extracted from the rhizomes of *Curcuma longa* Linn, also known as turmeric. It is the most abundant polyphenol and curcuminoid present in the root of this plant (7). Usually used in culinary and traditional medicine, curcumin also interests conventional medicine by demonstrating antioxidant and anti-inflammatory activities (7). Its anti-cancer effect was reported for the first time in 1985, by Kuttan and co-workers, in cells and animal models of lymphoma (8). In breast cancer, curcumin inhibits cell proliferation, induces apoptosis (5) and acts as a potent antiangiogenic, anti-invasive, and anti-metastatic agent *in vitro* and *in vivo* (9). Curcumin also demonstrated the capacity to reverse chemotherapeutic resistance in doxorubicin-resistant breast cancer cells (MCF-7/DOX and MDA-MB-231/DOX) (10). Moreover, curcumin led to the downregulation of aldehyde dehydrogenase-1, and p-glycoprotein-mediated multidrug resistance gene expression, increasing sensitivity of breast cancer cells (MCF-7) to paclitaxel (11). However, the therapeutic application of curcumin is limited due to its poor water solubility and low bioavailability. Thus, some studies have investigated the use of nanoparticles (NPs) to deliver curcumin to breast cancer cells and enhance its bioavailability and efficacy (12).

Nanotechnology is a strong alternative tool to improve application of hydrophobic molecules. The use of NPs increases the stability and bioavailability of antitumor compounds, reduces therapeutic doses, and minimizes possible adverse effects (13). Several types of NPs can be used as drug delivery systems, such as polymer NPs, liposomes, nanoemulsions, metal NPs, micelles, solid lipid NPs, dendrimers, nanospheres, and nanocapsules (14). These NPs can also be associated with other molecules like aptamers, antibodies, or polymers as active targeting moieties. This surface modification can improve the specificity of NPs to tumor cells, facilitate their interaction, and, consequently, increase antitumor effects (13).

At present, there are several clinical trials in which curcumin has been evaluated, mainly after oral administration regimens, in breast cancer patients (*e.g.*, NCT03980509, NCT01042938, NCT03847623, NCT03865992, NCT01740323, NCT01975363, NCT02556632, NCT01246973, NCT03482401) (15). So far, only one clinical trial using intravenous administration of a curcumin water-soluble formulation (CUC-1^®^) in combination with paclitaxel in breast cancer patients has been registered (NCT03072992) (15).

A substantial number of studies have been published describing the activity of Cur-NPs in *in vivo* models of breast cancer. Nevertheless, to the best of our knowledge, a systematic review on this subject has not been published yet. Therefore, considering the importance of *in vivo* studies and their clinical translation, the aim of this systematic review was to summarize published data regarding evaluations about efficacy and toxicity of Cur-NPs in *in vivo* models of breast cancer, as well as showing evidence for the potential of this therapeutic approach for clinical trial investigations. Although several works describing interesting data regarding the combination of curcumin with chemotherapeutic drugs have been published (16, 17), the present systematic review was performed to cover studies evaluating curcumin as the active antitumor agent, associated with NPs, to understand better the effects on breast tumor progression of curcumin itself and the advantages/limitations of using NPs as its dug carrier.

## Materials and Methods

### Protocol and Registration

The present study was conducted according to the Preferred Reporting Items for Systematic Reviews and Meta-Analyses (PRISMA) guidelines (18). The protocol for this systematic review was registered in the International Prospective Register of Systematic Reviews (PROSPERO) (19) with registration number: CRD42020209159.

#### Eligibility Criteria

##### Inclusion Criteria

This systematic review based the inclusion criteria on the PICOS (Population, Intervention, Comparison, Outcome, and Study Design) approach (20). We considered studies which evaluated the efficacy and toxicity (O) of Cur-NPs (I) compared with free curcumin and/or negative control (C) on *in vivo* models of breast cancer in mice or rats (P).

##### Exclusion Criteria

Studies were excluded for the following reasons: i) reviews, letters, personal opinions, book chapters, and conference abstracts; ii) *in vitro* studies and clinical trials; iii) use of only free curcumin or curcumin derivatives; iv) other types of cancer; v) use of Cur-NPs associated with other antitumor compounds; vi) full paper copy not available; vii) low quality.

#### Information Sources and Search Strategy

Individual search strategies were designed for each of the following bibliographic databases: CINAHL, Cochrane, LILACS, Embase, FSTA, MEDLINE, ProQuest, BSV regional portal, PubMed, ScienceDirect, Scopus, and Web of Science (**Table S1**). The search on databases was performed on August 10 and 11, 2020, with no time restriction. Duplicated references were removed by reference manager software (Mendeley^®^). There were no restrictions on language and period of publication.

#### Study Selection

The articles were selected in two phases: screening of titles and abstracts (phase 1) and full text reading (phase 2). In phase 1, two authors (A.S.O. and V.R.P.S) reviewed titles and abstracts of all references identified in the electronic databases and selected articles that seemed to meet the inclusion criteria. In phase 2, six pairs of authors (A.S.O and V.R.P.S.; L.R.A and G.J.F; W.O.P and A.C.P; J.V.O and M.P.G; M.S and G.F.G; P.M.C. and G.A.J.) were formed to independently analyze the full text of articles selected in phase 1 and exclude studies that did not meet the inclusion criteria (**Table S2**). A third author was consulted if disagreements between the two initial evaluators were not solved by consensus. Extraction of relevant data was done in all included studies to identify animal model, intervention (treatment regimen, dose, route, and NP platform) and outcomes (antitumoral activity and toxicity analysis).

#### Risks of Bias and Quality in Individual Studies

The quality of the articles included was estimated by applying the 10-question form from SYRCLE’s RoB Toll (**Table S3**) (21), to analyze the risk of selection, performance, detection, attrition, and other bias. The items were answered in each study by two reviewers individually and the disagreements were resolved by a third reviewer. YES answers indicated low risk of bias, NO indicated high risk of bias, and UNCLEAR indicated it was not possible to assign bias. As a secondary analysis, the quality of the studies was also assessed through 15 questions related to the methodology (**Table S4**) [based and adapted from the ARRIVE Guideline (22)], and measurement of the outcomes, which were raised by the reviewers and applied in order to classify the studies according to the percentage of YES responses to the criteria raised, being considered as high quality studies with > 70%, moderate quality with 50–69%, and low quality < 49%.

## Results

### Study Selection

Research on breast cancer has significantly increased. This can be observed, for example, when using the search string “(TITLE-ABS-KEY ((“Breast Cancer” OR “Breast Neoplasm” OR “Mammary Cancer” OR “Malignant Neoplasm of Breast” OR (“Mammary Carcinoma” AND Human) OR “Breast Carcinoma” OR “Cancer of Breast”)))”, on September 27, 2020, in the scientific database Scopus (23), for example, 530,186 document results were found. Of these, some of the regions/countries that published the most were: United States > China > United Kingdom > Germany > Italy > France > Japan > Canada > Australia > India, where the United States published 33.53% and China 8.32% of the studies.

**Figure 1:**
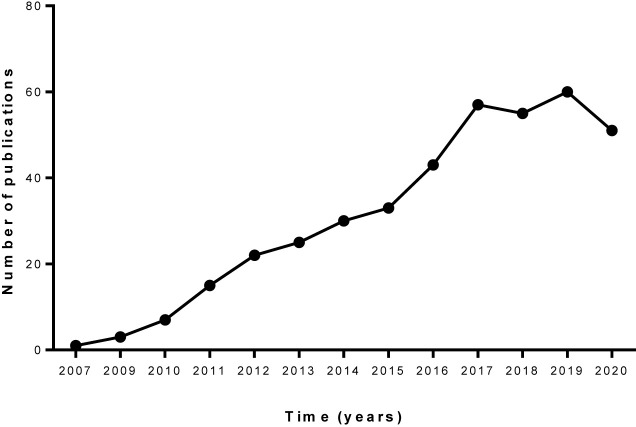
Number of publications listed in Scopus database with MeSH terms for curcumin nanoparticles and breast cancer. Data obtained on September 27, 2020.

When filtering these results with the search string “(TITLE-ABS-KEY ((“Breast Cancer” OR “Breast Neoplasm” OR “Mammary Cancer” OR “Malignant Neoplasm of Breast” OR (“Mammary Carcinoma” AND human) OR “Breast Carcinoma” OR “Cancer of Breast”) AND (curcumin OR “Turmeric Yellow” OR (yellow AND turmeric) OR diferuloylmethane)) AND TITLE-ABS KEY ((nanoparticles OR nanoparticle OR nanogels OR “Nanocomposite Gels” OR “Nanocomposite Gel” OR nanocapsule OR nanocapsules OR nanoemulsion OR micelle OR micelles OR liposome OR liposomal))),” in order to find studies that have specifically investigated curcumin associated with NPs, 402 documents were obtained (**Figure 1**). The countries/regions that most published on “breast cancer AND curcumin AND nanotechnology” were India, United States, China, Iran, Italy, among others, according to the Scopus database.

**Figure 2:**
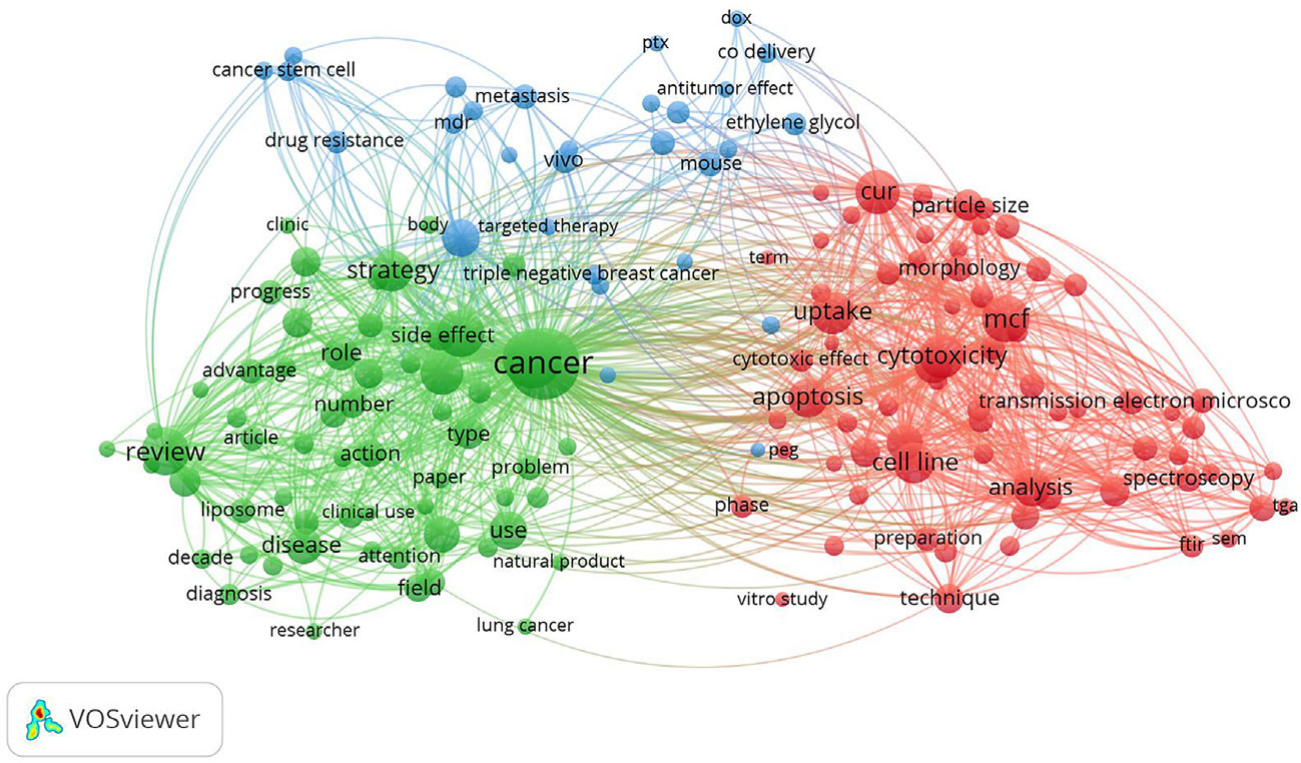
Bibliometric study of publications listed in Scopus database, with MeSH terms for curcumin nanoparticles and breast cancer, using the VOSviewer program (24), in “title and abstract”, with at least 10 co-occurrences and binary count configurations. Data obtained on September 27, 2020.

When conducting a bibliometric study of the results obtained in the last search in the Scopus database, using the VOSviewer 1.6.15 program (24), 159 terms were obtained, when establishing at least 10 co-occurrences and “binary count” (presence or absence in each study) configurations. **Figure 2** shows the interrelation of three clusters among the most recurrent terms used, where therapy, cytotoxicity, anticancer activity, and apoptosis were among the terms of greatest interest to the scientific community regarding the evaluated topic.

**Figure 3:**
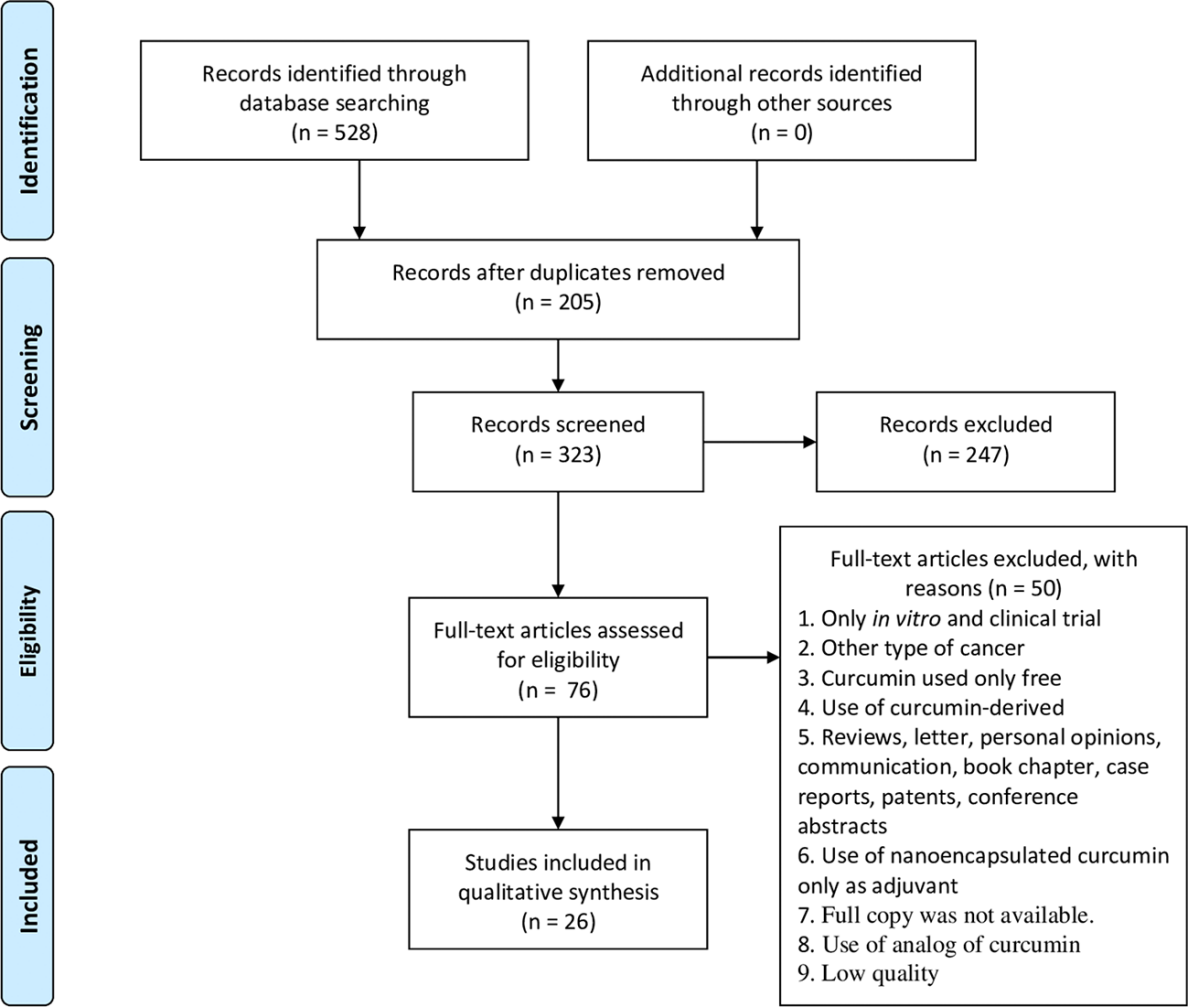
Flow diagram of literature search and selection criteria adapted from PRISMA (18).

Data presented in **Figures 1** and **2** were obtained using only the Scopus database in order to give a general overview of the topics discussed herein. However, in the present systematic review, a total of 528 studies were identified from different databases (57 from ScienceDirect; 35 from LILACS; 113 from Embase; 52 from MEDLINE; 60 from Portal Regional da BSV; 55 from PubMed; 41 from Web of Science; 112 from Scopus; 1 from CINAHL, 1 from FSTA, and 1 from ProQuest) (**Table S1**). After duplicate removal, 320 studies remained and an evaluation of “title and abstract” resulted in the exclusion of 244 studies. The remaining 76 articles were analyzed by full-text review. This process led to exclusion of 50 articles according to exclusion criteria (**Table S2**). In the end, 26 articles were maintained and included in this systematic review (**Table 1**) (25–29, 31–50). A flowchart detailing this process is shown in **Figure 3**.

**Table 1:** Summary of descriptive characteristics of the included studies.

Study	Population	Intervention			Outcomes	
Authors, year (reference)/country	Animal model^a^	Treatment regimen^b^	Dose; route	Nanostructure platform	Antitumor activity	Toxicity analysis
Shukla et al. (25)/India	Balb/c mice/n = 3/	Ten days from tumor inoculation;	100 mg/kg;	Lipid based CPC-SNEDDS NPs (Phospholipid, castor oil, Tween 80, PEG 400);	1) Cur-NP ↓TV (58.9%); free Cur ↓TV (29.5%); p<0.001	(ND)
	4T1/mouse/	Daily for 28 days	Oral	HD: 83.27 nm/PDI: 0.151;		
	(1 x 10^6^ cells)/	- Free Cur vehicle:		ZP: −16 mV/EE: 29.1%		
	subcutaneously on back skin	gum acacia (1%, w/v).				
Chen et al. (26)/China	Balb/c nude mice/n = 5/	TV of 200 mm^3^	5 mg/kg;	Micelles NPs [POCA4C6 (phosphorylated calixarene) micelles—PM];	1) Cur-NP ↓TV (~60%) and ↓ TW (~80%); free Cur: ↓TV (~34%) and ↓ TW (~60%); p<0.05	No damage in major organs;
	BT-549/human/	Fourteen days at every 2 days	Intratumoral	HD: 3.86 nm/PDI: 0.125;	2) Cur-NP ↑TNC and ↑TAP	No WL; hematological indices: ~ control
	(2 x 10^6^ cells)/	- Free Cur vehicle: (NM)		ZP: −25.18 mV/EE: 95.4%	3) Cur-NP ↓CD44+ CD133+ cancer stem cells	
	Subcutaneously on the upper right thigh					p<0.05
Mahalunkar et al. (27)/India, Germany, and Norway	Balb/c mice/n = 6/	First day of treatment: (NM)	10 mg/kg;	Metal gold NPs (CurAu-PVP) with folic acid (FA) (HAuCl_4_ and PVP polymer).	1) Cur-NP-FA ↓TV (~51%); free Cur: no ↓TV; p<0.006	(ND)
	4T1/mouse/	Twice a week for 2 weeks	Intratumoral	HD: 358.7 nm/PDI: 0.6	2) Cur-NP-FA ↓TW (~44%); free Cur: no TV↓; p<0.05	
	(1 x 10^5^ cells)/	- Free Cur vehicle: (NM)		ZP: −12.5 mV/EE: (NM)		
	Mammary fat pad					
Alizadeh et al. (28)/Iran	Balb/c mice/n = 8/	14 days after tumor induction;	Dose: (NM);	Micelles/polymersomes NPs (PNP) [monomethoxy-PEG (mPEG 2000), oleic acid (OA)]	1) Cur-NP ↓TV (~80%); p<0.05	31.25 mg/Kg of PNP-CUR: no damage in major organs;
	Transplantation of spontaneous mouse mammary tumor/	Daily for 24 days	Intraperitoneal	HD: 99.44 nm/PDI: 0.182;	2) Cur-NP ↑TAP; ↓ANG (CD31); ↓PROL (Ki-67); p<0.05	Hematological and biochemical indices: ~ control
	Pieces < 0.3 cm^3^/			ZP: −29.3 mV mV/EE: 64%		p<0.05
	Subcutaneous on the left flank					
Jung et al. (29)/Republic of Korea	Balb/c nude mice/n = 4/	TV of 50 mm^3^;	10 mg/kg;	Micelle NPs (DSPE-PEG micelle with or without EGFR specific targeting—EGF-Cur-NP)	1) Cur-NP-EGFR ↓TV (~59.1%); Cur-NP no ↓TV; p<0.05	No WL
	MDA-MB-468/human/	Three times a week; total of eight injections	Intraperitoneal	Cur-NP and EGF-Cur-NP: HD: 248.9 and 229.3 nm/PDI: 0.170 and 0.200, respectively		p<0.05
	(5 x 10^6^ cells)/			Cur-NP and EGF-Cur-NP: ZP: −3.6 and −1.73 mV, respectively/EE: (NM)		
	Right shoulder					
Wang et al. (30)/China	Nuke mice/n = (NM)/	Two months after tumor induction;	1 × 10^−3^ M;	Polymeric NPs (MPEG-PCL);	1) Cur-NP ↓TV (~82%); free Cur:↓TV (~49%); p<0.01	(ND)
	MDA-MB-231/human/	Daily for 2 weeks	Intravenous	HD: 139 nm/PDI: (NM);	2) Cur-NP ↑TAP	
	(1.5 x 10^6^ cells)/	- Free Cur vehicle: (NM)		ZP and EE: (NM)		
	Subcutaneous					
Laha et al. (31)/India and USA	Balb/c mice/n =6/	Ten days after tumor induction;	2 mg/kg (*unclear);	Metal organic frameworks NPs (IRMOF-3) with or without folic acid (FA) [(Zn (NO_3_)_2_; NH_2_-H_2_BDC]	1) Cur-NP-FA ↓TV (~61%); Cur-NP↓TV (~44%); p<0.05	Biochemical markers for liver and kidney: ~ control
	4T1/mouse/	Every 5 days for 4 times	Route of administration: (NM)	HD: 371,7 nm/PDI: 0.397	2) Cur-NP ↓TW (~74%); Cur-NP-FA ↓TW (~85%); p<0.05	p<0.05
	(NM)/			ZP: −10.9 mV/EE: 98%	3) Cur-NP and Cur-NP-FA ↓ tumor cell density	
	Mammary fat pad					
Vakilinezhad et al. (32)/Iran	Sprague Dawley rats/n = 6/	Four months after tumor induction;	2.5 mg;	Polymeric NPs (PLGA-PVA)	1) Cur-NP ↓TV (~20%); Free Cur: ↓TV (~16%); p<0.05	(ND)
	Chemically induced mammary tumors (MNU)	Once a week for 4 weeks	Intravenous	HD: 92.4 nm/PDI: 0.150		
		- Free Cur vehicle: aqueous suspension		ZP: −5.12 mV/EE: 89.4%		
Yuan et al. (33)/China	Balb/c nude mice/n = 6/	TV of 100 mm^3^;	2.5 mg/kg;	Polymeric NPs (mPEG- PLGA-Pglu)	1) Cur-NP ↓TV (~28.0%); p<0.05	No damage in major organs;
	MCF-7/human/	Every other day 4 times; total 18 d	Intravenous	HD: 228.5 nm/PDI: (NM)	2) Cur-NP ↓TW (~22.5%); p<0.05	No WL
	(3 x 10^6^ cells)/			ZP: −23.8 mV/EE: 76.9%	3) Cur-NP ↓breast cancer stem cells (~62%); p<0.05	p<0.05
	Right flank					
Sahne et al. (34)/Iran	Balb/c mice/n = 4/	TV of 50–100 mm^3^;	4 mg/kg;	Graphene oxide NPs (GO NPs with CMC, PVP, PEG, FA);	1) Cur-NP-FA↓TV (~86%); p<0.05	No damage in major organs;
	4T1/mouse/	Daily for a total 3 weeks	Intravenous	HD: ~60 nm/PDI: (NM)	2) Cur-NP-FA ↓TW (~76%); p<0.05	No WL
	(NM)/			ZP: −48 mV/EE: 94%	3) Cur-NP-FA ↑ ST and ↓ metastasis; p<0.05	p<0.05
	Subcutaneous on the flank				4) Cur-NP-FA: ↑TNC; ↓ cell density; ↓ANG (CD31, CD34); ↑ pro-inflammatory response in the tumor microenvironment; p<0.05	
Ji et al. (35)/China	Balb/c mice/n = 5/	First day of treatment: (NM);	5 mg/kg;	Nanocrystals NPs with or without HA	1) Cur-NP-HA ↓TV (~86%); Cur-NP↓TV (~39%); free Cur: ↓TV (~21%); p<0.05	No damage in major organs; no WL;
	4T1/mouse/	Every 2 days for a total 10 days	Intravenous	Cur-NP and HA-Cur-NP: HD: 101.4 and 161.9 nm/PDI: ~0.330 and 0.250, respectively	2) Cur-NP-HA ↓TW (~75%); Cur-NP↓TW (~37.5%); Free Cur: ↓TW (~25%); p<0.05	No hemolysis (<5%);
	(1 x 10^6^ cells)/	- Free Cur vehicle: (NM)		HA-Cur-NP: ZP: −25.0 mV, respectively/EE: (NM)	3) ↑ ST: Cur-NP-HA > Cur-NP > Free Cur; p<0.05	Hematological and biochemical indices: ~ healthy control
	Subcutaneous on the right flank					p<0.05
He et al. (36)/China	Balb/c mice/n = 6/	TV of 100 mm^3^	5mg/kg;	Polymeric micellar NPs [amphiphilic diblock copolymer— mPEG-b-PLG (Se)-TP];	1) Cur-NP ↓TV (~65%); Free Cur: ↓TV (~49%); p<0.05	No damage in major organs; no WL
	4T1/mouse/	Every 4 days, for 4 times; total 21 days	Intravenous	HD: 136 nm/PDI: 0.071	2) Cur-NP ↓TW (~82%); free Cur: ↓TW (~62%); p<0.05	p<0.05
	(1 x 10^6^ cells)/	- Free Cur vehicle: (NM)		ZP: (NM)/EE: ~ 68%	3) Cur-NP ↑TNC and ↑TAP; ↓ANG (CD31); ↓PROL (Ki-67); p<0.05	
	Subcutaneous on the right back					
Jin et al. (37)/China and USA	Balb/c nude mice/n = 5/	7 days after tumor induction;	5 mg/kg;	Polymeric NPs with or without EGFR-targeting peptides (GE11) (PLGA-PEG);	1) Cur-NP-GE11 and Cur-NP ↓TV (~80%); free Cur: no TV↓; p<0.05	Inflammatory cytokine levels: ~ healthy mice
	MCF-7/human/	Every 24 h for 20 times	Intravenous	HD: 210 nm/PDI: 0.112;	2) Cur-NP-GE11↓TW (~56%); Cur-NP ↓TW (~48%); free Cur: no TW↓; p<0.05	p<0.05
	(1 x 10^7^ cells)/			ZP: −22 mV/EE: 92.3	3) Cur-NP-GE11and Cur-NP ↑TAP	
	Subcutaneous on the dorsal flank	- Free Cur vehicle: (NM)				
Abd-Ellatef et al. (38)/Italy and Egypt	Balb/c mice/n = 8/	TV of 50 mm^3^;	5 mg/kg;	Solid lipid nanoparticles (SLN) with or without chitosan (CS) coating (cholesterol; trilaurin, butyl lactate, Epikuron^®^200, Cremophor^®^RH60, sodium taurocholate, Pluronic^®^ F68);	1) Cur-NP-CS and Cur-NP ↓TV (~35%); Free Cur: no TV↓; p<0.01	Biochemical indices: ~ control
	JC/mouse/	Thrice (on day 1^st^, 7^th^, 14^th^)	Intravenous	HD: < 200 nm/PDI: (NM)		p<0.05
	(1 x 10^7^ cells)/	- Free Cur vehicle: 10% v/v DMSO suspension		ZP: (NM)/EE: 70–75%		
	Mammary fat pad					
Li et al. (39)/China	Balb/c mice/n = 4/	Tumor diameter of 4 mm;	8 mg/kg;	Mesoporous silica nanoparticles with hyaluronan (MSN-HA) or polyethyleneimine-folic acid (MSN-PEI-FA).	1) Cur-NP-PEI-HA ↓TV (~50%); Free Cur: no TV↓; p<0.01	No damage in major organs; no WL;
	MDA-MB-231/human/	Every 3 days for a total of six times	Intravenous	HD: < 300 nm/PDI: (NM)/	2) Cur-NP-PEI-HA ↓TW (~70%); free Cur: no TW↓; p<0.01	Hemolysis (<5%);
	(1 x 10^7^ cells)/	- Free Cur vehicle: (NM)		ZP: ~ −20 mV (MSN-HA); ~ +40 mV (MSN-PEI-FA)		Biochemical indices: ~ healthy control
	Subcutaneous					p<0.05
Kundu et al. (40)/India	Swiss albine mice/n = 6/	Ten days after induction;	10 mg/kg;	Metal NPs [Zinc oxide nanoparticles (ZnO) with PBA];	1) Cur-NP↓TV (~77%); free Cur: ↓TV (~66%); p<0.05	No damage in the liver and kidney;
	Ehrlish ascites carcinoma cells/	Alternate days for 14 days.	Intravenous	HD: 413.63 nm/PDI: (NM)	2) Cur-NP ↓TW (~72%); free Cur: ↓TW (~50%); p<0.05	Biochemical markers: ~control; ↓ tumor-induced splenomegaly
	(1.0 x 10^7^/ml)	- Free Cur vehicle: (NM)		ZP: −16.4 mV/EE: 27%	3) Cur-NP and free Cur: ↑TAP; p<0.05	p<0.05
	Left flank					
Lv et al. (41)/China	Kunming (mice)/n = 6/	TV of 300 mm^3^;	10 mg/kg;	Polymeric NPs (PEG-PCDA) with or without biotin;	1) Cur-NP ↓TV (~69%); Cur-NP-biotin ↓TV (~79%); free Cur: ↓TV (~32%); p<0.05	no WL
	EMT6/mouse/	Daily for 9 days; total 14 days	Intravenous	PEG-PCDA and biotin-PEG-PCDA: HD: 94.2 and 125.1 nm/PDI: 0.170 and 0.08, respectively	2) Cur-NP ↓TW (~70%); Cur-NP-biotin ↓TW (~85%); free Cur: ↓TW (~25%); p<0.05	p<0.05
	(1.0 x 10^7^/ml)	- Free Cur vehicle: cremophor EL: dehydrated alcohol (1:1, v/v) and diluted with physiological saline		PEG-PCDA and biotin-PEG-PCDA: ZP: −9.56 and −12.86 mV/EE: (NM)	3) Cur-NP and Cur-NP-biotin: ↑TNC and ↑TAP; ↓ANG (CD31; VEGF; COX-2); ↓PROL (Ki-67); p<0.05	
	Subcutaneously					
Yang et al. (42)/China	Balb/c nude mice/n = 5	TV of 200 mm^3^	10 mg/kg;	Micelle NPs (triblock copolymer PPBV);	1) Cur-NP ↓TV (~58.5%, day 12); ↓TV (~28.9%, day 20); p<0.05	No WL
	MCF-7/human/	Every other day for 5 times; total 20 days	Intravenous	HD: 6.7 nm/PDI: 0.117	2) Cur-NP ↓TW (~22%, day 20); p<0.05	p<0.05
	(1 x 10^7^ cells)/			ZP: −1.42 mV/EE: 68.5%		
	Subcutaneous on the flank	- Free Cur vehicle: (NM)				
Yang et al. (43)/China	Balb/c nude mice/n = 5/	TV of 200 mm^3^	15 mg/kg;	Hybrid NPs [PLGA NPs coated with a modified hialuronic acid (HA-hybrid)]	1) Cur-NP-HA ↓TV (~43.8%, day 12); ↓TV (~24%, day 20); p<0.05	No WL
	MCF-7/human/	Every other day for 5 times; total 20 days	Intravenous	HD: 350 nm/PDI: (NM)	2) Cur-NP-HA ↓TW (~22%, day 20); p<0.05	p<0.05
	(1 x 10^7^ cells)/			ZP: −22 mV/EE: 32%	3) Cur-NP-HA ↓tumor cell density; p<0.05	
	Subcutaneous on the flank	- Free Cur vehicle: (NM)				
Greish et al. (44)/Bahrain	Balb/c mice/n = 5/	TV of 100 mm^3^;	10 and 20 mg/kg;	Micelles (curcumin–metal complex and SMA)	1) Cur-NP-10mg/Kg ↓TV (~61%); Cur-NP-20mg/Kg ↓TV (~92%); p<0.05	(ND)
	4T1/mouse/	frequency of treatment: unclear; total 10 days	Intravenous	HD: 248 nm/PDI: 0.274;		
	(1 x 10^6^ cells)/			ZP: −11 mV/EE: 80%		
	Bilaterally on the flanks					
Mukerjee et al. (45)/USA	Balb/c nude mice/n = 8/	TV of 70 mm^3^;	20 mg/kg;	Polymeric NPs [PLGA/PVA with or without antibody targeting (AnxA2)]	1) Cur-NP- AnxA2 ↓TV (~44.0%); Cur-NP ↓TV (~33.5%); p<0.05	No WL
	MCF10CA1a/human/	Thrice week for a total 30 days	Intravenous	Cur-NP and AnxA2-Cur-NP: HD: 150 and 157 nm/PDI: ~0.240 and 0.200, respectively	2) Cur-NP- AnxA2 ↓TW (~53.0%); Cur-NP ↓TW (~30%); p<0.05	p<0.05
	(3 x 10^6^ cells)/			Cur-NP and AnxA2-Cur-NP: ZP: −27.5 and −28.5 mV, respectively/EE: 89.2%	3) Cur-NP- AnxA2 and Cur-NP: ↓ANG; ↓ NFkβ; ↓PROL (Ki-67); p<0.05	
	Flank					
Mukhopadhyay et al. (46)/India	Balb/c nude mice/n = 5/	8 days after induction;	20 mg/kg	Polymeric NPs [PLGA/PVA with or without folate (F)]	1) Cur-NP-F ↓TV (~90%); Cur-NP↓TV (~75%); p<0.05	No damage in major organs
	MDA-MB-231/human/	Thrice week for a total 21 days	Route of administration: unclear	HD: 170 nm/PDI: 0.186;	2) Cur-NP-F ↓TW (~92%); Cur-NP↓TW (~61.5%); p<0.05	p<0.05
	(5 x 10^6^ cells)/			ZP: −28.2 mV/EE: 68.6%	3) Cur-NP-F and Cur-NP ↓ cell density	
	Right flank					
Yu et al. (47)/China	Balb/c nude mice/n = 5/	TV of 100–400 mm^3^;	40 mg/kg	Micelles NPs (MPEG-PLA with or without PAE)	1) Cur-NP-PAE ↓TV (~65.6%); Cur-NP ↓TV (~47.1%); p<0.05	no WL
	MCF-7/human/	Every other day for 5 times for a total 24 days	Intravenous	HD: 128.4 nm to 171.0 nm/ PDI: 0.118 to 0.134	2) Cur-NP-PAE ↓TW (~76%); Cur-NP ↓TW (~53%); p<0.05	p<0.05
	(3 x 10^6^ cells)/			ZP: −2.0 to +4.0 mV/ EE: 96.5 to 98.8%		
	Subcutaneously right flank					
Huang et al. (48)/China	Balb/c mice/n = 5/	TV of 40–50 mm^3^/	50 mg/kg	Polymeric NPs (HA-CHEMS); pH-sensitive	1) Cur-NP ↓TV (~38%); p<0.05	↓ Damage in major organs
	4T1/mouse/	Every 2 days for a total of 5 times	Intravenous	HD: 144 nm/PDI: (NM);	2) Cur-NP ↑ ST;	no WL
	(NM)/			ZP: −21.25 mV/EE: (NM)	3) Cur-NP ↑TNC and ↑TAP	p<0.05
	Flank					
Shiri et al. (49)/Iran	Balb/c mice/n = 9/	Third day after tumor induction/	40 mg/kg or 80 mg/kg	Dendrosome NPs (DNC) [composition: not mentioned (patent number: 71753)]	1) NP-40mg/Kg ↓TV (~72%); NP-80mg/Kg ↓TV (~76%); p<0.05	no WL
	4T1/mouse/	daily for 35 consecutive days	Route of administration: (NM)	HD; PD; ZP; EEI: (NM)	2) NP-40mg/Kg ↓TV (~61%); NP-80mg/Kg ↓TV (~64%); p<0.05	No change in food intake and behavior
	(1 x 10^6^ cells)/				3) NP ↑ ratio of M1/M2 macrophages	p<0.05
	left flank					
Lin et al. (50)/China	Balb/c nude mice/n = 6/	First day of treatment: (NM)/	Dose: (NM)/	Lipid based NPs (NLC) with or without folate (FA) coating (PEG-DSPE, soya lecithin, castor oil, Tween 80, Precirol ATO-5);	1) Cur-NP-FA ↓TV (~83%); Cur-NP ↓TV (~66%); free Cur: ↓TV (~31%);	No WL
	MCF-7/human/	once every 3 days for 15 days	Intravenous	HD: 126.8 nm/PDI: 0.16		p<0.05
	(NM)/			ZP: +12.6 mV/EE: 82.7%		
	Subcutaneous in the right armpit	- Free Cur vehicle: (NM)				

a Animal type/replicates/cell type injected/source/cell concentration/local of cell insertion;

b First day of treatment (or tumor volume)/treatment frequence and total experiment time; ANG, angiogenesis; CHEMS, cholesteryl hemisuccinate; CMC, carboxymethyl cellulose; CPC-SNEDDS, curcumin-phospholipid complex self-nanoemulsifying drug delivery systems; Cur, curcumin; d, days; DSPE, distearoyl phosphatidyl- ethanolamine; EGFR, epidermal growth factor receptor; FA, folic acid; HA, hyaluronic acid; MNU, N-methyl nitroso urea; mPEG-b-PLG (Se)-TP, poly-(ethylene glycol)-b-poly(L-glutamic acid)-two-photon AIE fluorophores [mPEG-b-PLG (Se)-TP] amphiphilic copolymer with selenide group (Se) conjugated and a two-photon AIE fluorogen (TP) on the terminal group of PLG segments.; MPEG-PCL, methoxypoly(ethylene glycol)-polycaprolactone; NF-κB, nuclear factor kappa b; NH2-H2BDC, 2-amino terephthalic acid; (ND), not determined; (NM), not mentioned; NPs, nanoparticles; PAE, poly (b-aminoester; PBA, phenyl boronic acid; PCDA, pentacosadiynoic acid; PEG, poly(ethylene glycol); PGlu, poly L-glutamic acid; PLA, polylactic-co-glycolic acid; PPBV, mPEG-PBLA-PVIm triblock copolymer; PROL, tumor proliferation; PVA, polyvinyl alcohol; PVP, polyvinylpyrrolidone; SMA, poly(styrene)-co-maleic acid; ST, survival time; TAP, tumor apoptosis; TNC, tumor necrosis; TV, tumor volume; TW, tumor weight; WL, weight loss.

### Characteristics of the Included Studies

All included studies are research articles that evaluated antitumoral activity of Cur-NPs in *in vivo* models of breast cancer. The main characteristics of the studies are summarized in **Table 1**.

The studies were conducted in several countries: China (n = 13); India (n = 6); Bahrain (n = 1); Iran (n = 4); Italy (n = 1); US (n = 1); Republic of Korea (n = 1), and all of them were published from 2014 to 2020 in the English language.

Cur-NPs used in the included studies were mainly described by hydrodynamic diameter (HD) (n=26), polydispersity index (PdI) (n=17), and zeta potential (n=21) through dynamic light scattering and electrophoretic mobility. Some studies assessed NPs’ size and/or morphology by transmission electron microscopy (TEM) and/or scanning electron microscopy (SEM). Curcumin encapsulation efficiency (EE%) was evaluated mostly by high-performance liquid chromatography (HPLC) (n=18).

All *in vivo* studies followed the progression of tumor volume during the experimental period by measuring tumor small/large diameters or width/length and calculating the final volumes with mathematical formulas. Studies also assessed tumor weight (n=16); survival time (n=3); tumoral stem cells through flow cytometry (n=2); ratio of M1/M2 macrophages through RT-PCR (n=1); apoptosis (n=8), necrosis (n=5), proliferation (n=4), angiogenesis (n=5), cell density (n=4), inflammatory response in the tumor (n=1), and metastasis (n=1) through classical histology (HE) and immunohistochemistry. Regarding toxicity analysis, 21 studies used at least one parameter of evaluation. Studies assessed weight loss (n=15); damage of major organs through classical histology (HE) (n=9); food intake/behavior (n=1); inflammatory cytokine levels (n=1) through ELISA; hemolysis (n=2) through absorbance; and hematological (n=3) and biochemical parameters (n=6) through animal blood counter and commercial kits.

### Quality and Risk of Bias in Individual Studies

When analyzed by questions based on ARRIVE guidelines, as seen in **Table 2**, 23 studies were graded as of high quality and 3 as of moderate quality. Most studies clearly reported the encapsulation methods of curcumin (n=24) and investigated characteristics of NPs (n=23). Animal models were considered adequate in all studies and ethical committee approval was clearly reported in 18 studies. Six studies did not clearly report ethical approval and two other articles did not mention this criterion. Furthermore, animal conditions during the experiment were not clearly described in 13 studies. The study design of anticancer activity was well executed in most studies and some articles clearly mentioned time of treatment (n=25), route of administration (n=22), dose of curcumin (n=25), and presence of control groups (n=25). Nevertheless, only 15 studies investigated anticancer activity of free curcumin to compare with the Cur-NP effect. Additionally, a toxicity assay was performed in 21 studies. Lastly, statistical models were considered as unclear in all studies due to lack of information.

**Table 2:** Overall quality of the selected studies. Detailed description of the evaluated parameters is found in **Table S4**.

Critical analysis of the articles included																		
Author	Year	Methodology											Toxicity		Outcomes		Score	Classification
		1	2	3	4	5	6	7	8	9	10	11	12	13	14	15	%	
Abd-Ellatef et al. (38)	2020	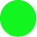	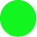	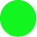	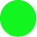	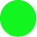	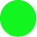	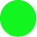	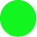	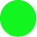	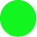	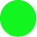	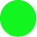	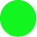	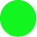	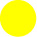	93	High quality
Alizadeh et al. (28)	2015	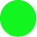	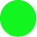	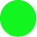	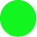	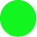	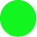	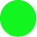	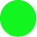	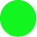	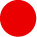	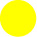	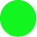	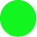	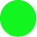	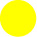	80	High quality
Chen et al. (26)	2017	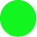	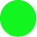	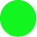	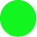	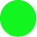	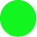	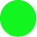	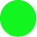	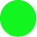	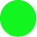	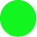	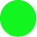	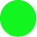	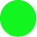	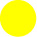	93	High quality
Greish et al. (44)	2018	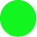	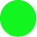	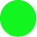	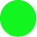	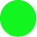	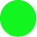	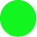	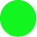	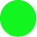	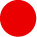	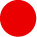	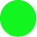	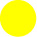	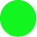	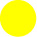	73	High quality
He et al. (36)	2019	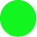	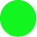	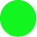	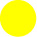	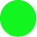	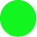	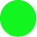	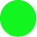	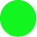	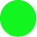	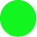	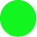	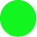	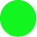	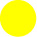	86	High quality
Huang et al. (48)	2020	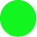	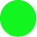	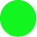	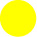	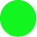	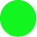	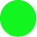	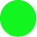	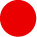	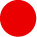	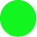	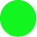	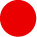	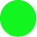	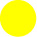	66	Moderate quality
Ji et al. (35)	2020	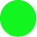	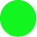	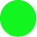	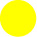	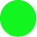	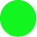	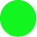	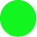	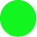	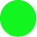	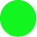	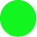	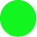	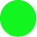	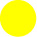	86	High quality
Jin et al. (37)	2017	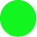	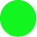	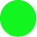	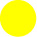	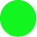	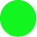	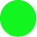	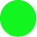	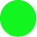	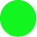	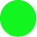	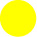	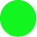	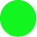	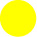	86	High quality
Jung et al. (29)	2018	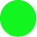	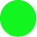	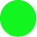	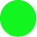	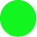	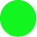	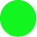	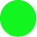	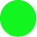	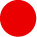	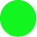	-	-	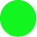	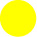	86	High quality
Kundu et al. (40)	2019	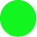	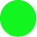	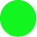	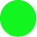	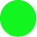	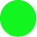	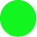	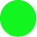	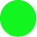	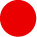	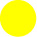	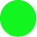	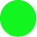	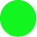	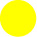	80	High quality
Laha et al. (31)	2019	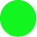	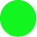	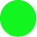	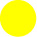	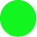	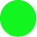	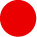	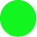	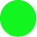	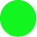	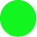	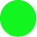	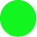	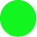	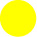	73	High quality
Li et al. (39)	2018	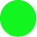	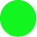	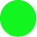	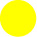	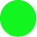	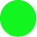	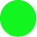	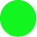	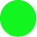	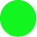	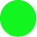	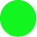	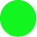	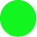	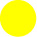	86	High quality
Lin et al. (50)	2016	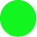	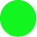	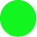	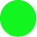	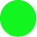	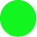	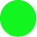	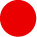	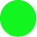	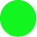	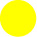	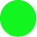	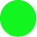	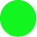	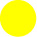	80	High quality
Lv et al. (41)	2015	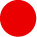	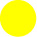	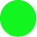	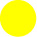	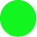	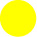	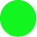	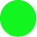	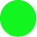	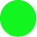	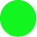	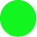	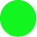	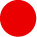	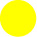	60	Moderate quality
Mahalunkar et al. (27)	2019	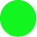	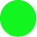	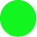	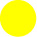	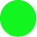	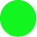	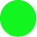	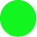	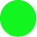	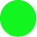	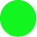	-	-	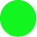	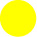	86	High quality
Murkerjee et al. (45)	2016	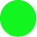	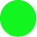	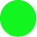	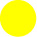	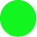	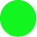	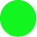	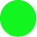	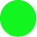	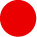	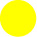	-	-	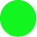	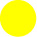	73	High quality
Mukhopadhyay et al. (46)	2020	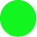	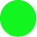	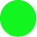	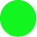	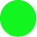	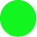	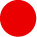	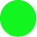	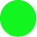	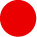	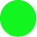	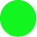	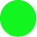	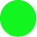	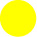	80	High quality
Sahne et al. (34)	2019	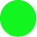	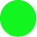	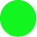	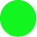	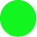	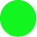	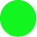	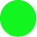	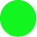	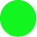	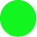	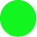	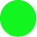	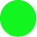	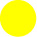	93	High quality
Shiri et al. (49)	2015	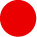	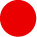	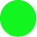	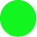	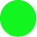	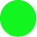	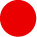	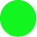	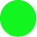	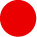	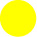	-	-	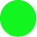	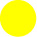	53	Moderate quality
Shukla et al. (25)	2017	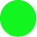	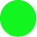	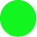	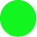	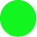	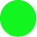	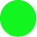	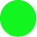	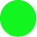	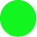	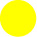	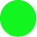	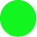	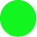	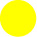	86	High quality
Vaklinezhad et al. (32)	2019	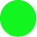	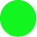	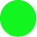	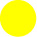	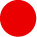	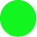	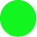	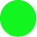	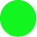	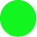	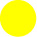	-	-	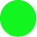	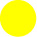	73	High quality
Wang et al. (30)	2018	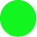	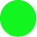	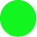	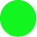	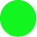	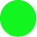	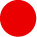	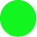	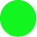	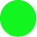	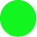	-	-	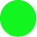	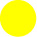	86	High quality
Yang et al. (42)	2017a	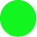	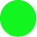	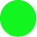	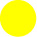	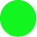	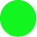	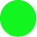	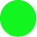	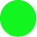	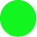	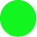	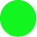	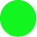	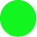	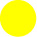	86	High quality
Yang et al. (43)	2017b	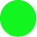	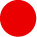	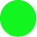	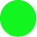	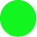	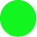	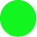	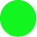	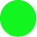	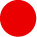	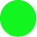	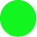	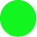	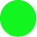	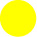	80	High quality
Yu et al. (47)	2014	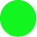	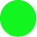	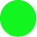	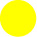	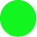	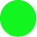	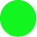	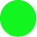	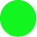	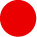	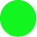	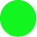	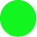	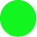	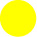	80	High quality
Yuan et al. (33)	2018	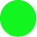	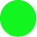	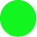	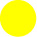	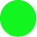	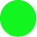	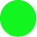	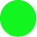	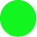	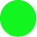	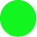	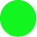	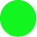	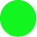	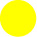	86	High quality

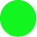
 Yes 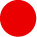 No 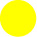 Unclear - Not evaluated.

Risk of bias assessment based on SYRCLEs’ RoB guidelines of all included studies is summarized in **Table 3**. Criteria were considered unclear when they were not clearly reported or gave incomplete information. Most studies did not clearly describe information on allocation, randomization, and blinding, which is required for quality assessment. Animals were not randomly housed during the experiment in two studies. Additionally, three studies did not adequately address incomplete outcome data. In parallel, the experimental groups were considered similar in 18 studies, and 24 studies showed low risk of selection bias of reported outcomes.

**Table 3:** Risk of bias in individual studies (SYRCLE’s Rob toll criteria). YES answers indicated low risk of bias, NO indicated high risk of bias, and UNCLEAR indicated it was not possible to assign bias. Detailed description of the evaluated parameters is found in **Table S3**.

SYRCLE’s Risk of Bias											
Author	Year	Selection			Performance		Detection		Attrition	Reporting	Other
		1	2	3	4	5	6	7	8	9	10
Abd-Ellatef et al. (38)	2020	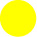	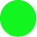	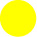	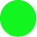	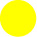	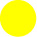	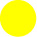	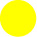	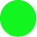	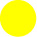
Alizadeh et al. (28)	2015	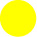	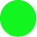	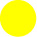	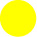	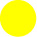	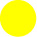	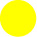	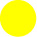	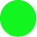	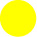
Chen et al. (26)	2017	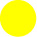	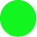	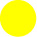	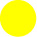	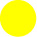	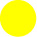	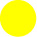	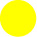	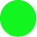	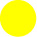
Greish et al. (44)	2018	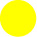	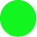	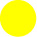	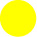	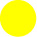	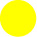	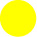	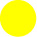	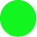	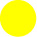
He et al. (36)	2019	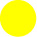	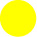	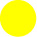	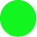	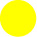	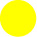	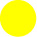	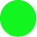	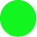	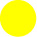
Huang et al. (48)	2020	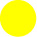	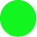	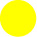	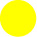	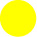	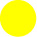	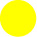	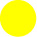	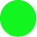	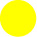
Ji et al. (35)	2020	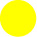	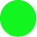	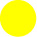	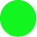	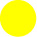	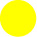	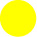	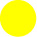	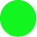	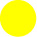
Jin et al. (37)	2017	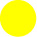	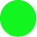	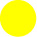	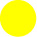	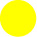	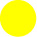	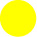	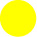	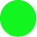	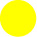
Jung et al. (29)	2018	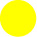	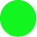	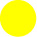	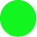	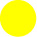	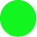	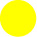	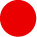	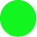	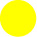
Kundu et al. (40)	2019	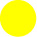	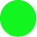	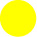	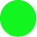	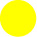	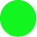	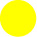	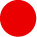	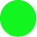	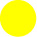
Laha et al. (31)	2019	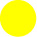	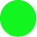	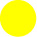	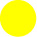	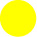	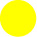	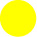	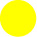	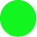	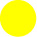
Li et al. (39)	2018	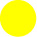	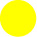	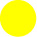	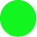	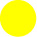	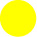	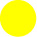	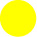	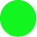	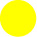
Lin et al. (50)	2016	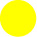	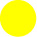	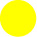	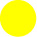	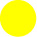	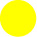	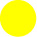	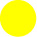	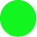	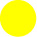
Lv et al. (41)	2015	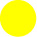	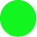	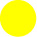	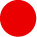	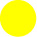	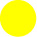	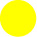	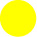	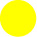	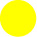
Mahalunkar et al. (27)	2019	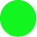	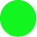	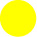	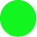	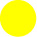	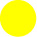	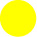	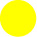	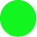	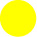
Murkerjee et al. (45)	2016	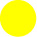	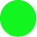	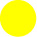	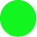	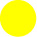	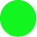	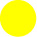	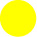	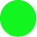	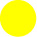
Mukhopadhyay et al. (46)	2020	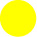	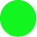	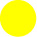	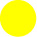	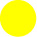	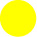	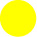	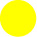	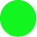	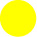
Sahne et al. (34)	2019	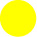	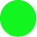	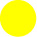	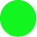	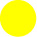	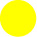	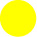	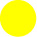	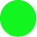	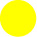
Shiri et al. (49)	2015	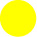	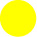	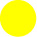	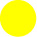	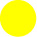	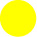	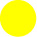	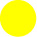	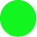	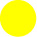
Shukla et al. (25)	2017	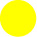	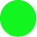	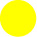	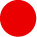	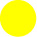	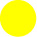	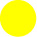	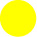	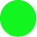	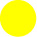
Vaklinezhad et al. (32)	2019	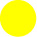	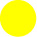	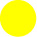	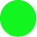	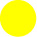	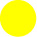	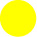	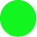	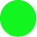	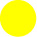
Wang et al. (30)	2018	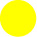	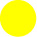	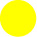	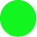	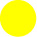	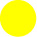	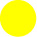	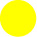	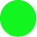	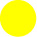
Yang et al. (42)	2017a	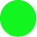	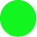	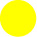	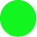	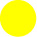	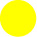	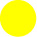	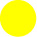	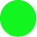	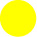
Yang et al. (43)	2017b	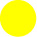	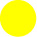	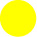	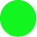	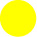	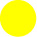	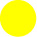	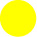	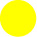	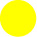
Yu et al. (47)	2014	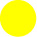	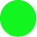	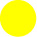	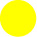	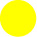	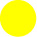	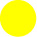	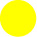	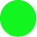	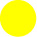
Yuan et al. (33)	2018	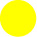	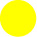	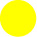	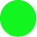	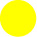	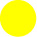	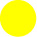	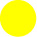	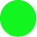	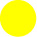

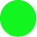
 Yes 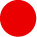 No 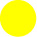 Unclear - Not evaluated.

### Synthesis of Results

A wide variety of NP types were used in the included studies. Polymer NPs were mostly used for curcumin delivery (n = 9), followed by micelles (n = 6), lipid-based NPs (n=3), metal NPs (n = 2), hybrid NPs (n=2), dendrosomal NP (n =1), nanocrystal (n=1), graphene oxide NP (n=1), and mesoporous silica NP (n=1). Poly(ethylene glycol) chains (PEG) were present in NP composition in 11 studies. Nine studies evaluated NPs associated with targeting moieties such as folic acid (n=5), hyaluronic acid (n=1), EGF peptides (n=2), and AnxA2 (n=1). With HD ranging from 101.4 to 371.7 nm, most Cur-NPs presented negative Zeta potential (−48 to +40 mV) (n=22) and EE% from 32 to 98% (**Table 1**).

Concerning experimental design, studies were heterogeneous regarding animal model, route of administration, duration of the experiment, and dose of treatment. Ten studies adopted nude mice as animal models when tumor was induced with human cells and another 16 used mice with a native immune system for tumor induction with murine cells. One study used rats with MNU-chemically induced mammary tumors (n=1). The main human cell lines used were MCF-7 (n=6), MDA-MB-231 (n=3), MDA-MD-468 (n=1), MCF10CA1a (n=1), and BT-549 (n=1). The murine cell lines used were 4T1 (n=9), EMT6 (n=1), JC (n=1), and Ehrlich ascites carcinoma cells (n=1). One study used the transplantation of spontaneous mouse mammary tumor pieces (n=1) as the breast cancer model. Implantation of tumor cells into the mammary fat comprised three studies, while the others adopted subcutaneous implantation into the flank (n=14) or armpit (n=1).

The first day of treatment was described according to days after induction (n=9), ranging from 3 days to 4 months, or tumor volume (n=13) in the range of 40 to 400 mm^3^. The curcumin doses used in the treatments varied between 2 and 100 mg/Kg and were administered daily (n=8), every other day (every 2 days) (n=7), three times a week (n=6); twice a week or less (n=4). Intravenous administration was the main route of administration used in the included studies (n=18). Few studies used intraperitoneal (n=2), intratumoral (n=2), or oral administration route (n=1); and one did not clearly mention this information (n=1).

## Discussion

### Summary of Evidence

The structure of curcumin has chemical groups that allow interactions of diverse chemical natures (*e.g.*, covalent, non-covalent, hydrophobic, and hydrogen bonds) with molecules involved in the different pathways of breast carcinogenesis (9, 16). It has been reported that curcumin inhibits cell proliferation, tumor invasion, and angiogenesis. As an anti-proliferative agent, curcumin induces cell cycle arrest and p53-dependent apoptosis. It also alters signaling protein expression, such as Ras, protein kinase B (Akt), and phosphatidylinositol-3-kinase (PI3K) (51). Additionally, the use of curcumin has been described as a potential strategy for inhibiting EZH2 (enhancer of zeste homolog-2), a histone modifier protein subunit involved in tumor growth, metastatic potential, and in the regulation of drug resistance. In breast cancer, it has been reported that curcumin is able to inhibit the proliferation of human breast cancer MDA-MB-435 cells in correlation with the downregulation of EZH2 expression (52, 53).

Curcumin has demonstrated anti-invasive effects through downregulation of matrix metalloproteinase (MMP-2) and upregulation of tissue inhibitor of metalloproteinase (TIMP-1) in MDA-MB-231 breast cancer cells (54). Interestingly, emerging evidence indicates that the chemopreventive and chemotherapeutic properties of curcumin are closely linked to the modulation of miRNAs involved in tumorigenesis and metastasis signaling pathways, *e.g.*, hedgehog, notch-1, PI3K/Akt/mTOR, Wnt/β-catenin, IGF, VEGF, and TGF-β/smad3 pathways (55, 56). Gallardo et al. (57) demonstrated that curcumin prevents the migration and invasion of breast cancer cells (MCF-10F and MDA-MB-231) by targeting miR-34a as a regulator of *Rho-A* and other genes involved in epithelial-mesenchymal transition, such as *Axl, Slug*, and *CD24* (57). Curcumin can also prevent angiogenesis by inhibiting vascular endothelial growth factor (VEGF) (58, 59) and suppressing angiogenic cytokine interleukin-6 (60). All of the mentioned mechanisms cited above along with anti-inflammatory action and inhibition of cell growth factors, support confirmation of the wide activity of curcumin in the regulation of tumor growth by acting in different cancer hallmarks (9).

However, the hydrophobic property of curcumin limits its applications and demonstrates less impressive success in clinical trials (14). Additionally, free curcumin can undergo biomodifications and may be mostly excreted in feces or in bile in animal models (61). Therefore, the use of drug delivery systems, such as NP platforms, is an alternative to improve drug bioavailability, administer lower doses, increase time of circulation, and enhance its biological activity. The natural product-based nanomedicine field for cancer treatment has increased and demonstrated great potential (6, 14). The present systematic review reports the effects of Cur-NPs on antitumoral activity and toxicity of in *in vivo* models of breast cancer.

Analysis of the included studies showed that curcumin evaluated in *in vivo* models of breast cancer has been loaded in a wide variety of NP platforms, including different compositions, sizes, and zeta potential. In fact, advantages were evidenced in all the included studies that compared tumor volume reduction after Cur-NP treatment with curcumin treatments in its free form and/or with negative controls (**Table 1**). For instance, a volume reduction of ~21% was observed in tumors of animals treated with free curcumin, while a significant reduction of ~86% was observed in animals treated with curcumin nanocrystals coated with hyaluronic acid (35).

NPs are able to accumulate into solid tumors (*e.g.*, breast cancer). The classical concept states that NPs extravasate the tumor’s vascular barrier through gaps between endothelial cells (owing to irregular angiogenic growth) and are retained in the tumor mass due to poor local lymphatic drainage—a passive process known as the enhanced permeability and retention (EPR) effect (62, 63) Nevertheless, this pathway has been currently under debate, and updated data show evidence that it may not be the dominant mechanism of NPs’ extravasation into solid tumors (64, 65). Other mechanisms of NPs’ tumor accumulation have been investigated, such as the trans-endothelial pathway, which is a metabolically active process that requires endothelial cells to rearrange their structure to present vesicles that can uptake NPs and further deliver them to tumor cells nearby (64).

Improvements in tumor NP accumulation and favorable interaction between NPs and cancer cells can be obtained by tailoring the surface of the NP with moieties able to confer prolonged blood-circulation time (*e.g.*, PEG) and specific active targeting (*e.g.*, ligands with affinity to molecules overexpressed in tumor cells) (66). The main active targeting moieties found in the included studies were folic acid (FA) and hyaluronic acid (HA). FA shows affinity to folate receptors, which are tumor-associated proteins overexpressed in more than 40% of human tumors, including breast cancer (67). A metal organic framework of FA-Cur-NPs significantly improved curcumin antitumor efficacy (~61%), while non-modified NP accounted for ~44% for tumor volume reduction (31). Similarly, attachment of HA, a natural polysaccharide consisting of repeating disaccharide units, to the surface of NPs, has been investigated since it binds to the cell surface molecule CD44, a surface protein widely expressed in breast cancer (68). Curcumin associated with HA-mesoporous silica NPs showed a significant ~70% reduction in tumor weight, while no significant effect was observed for free curcumin (39). Other modifications of the curcumin-NP surface with ligands specific to different tumor surface biomarkers for breast cancer have also been explored (29, 37, 45).

Once in the tumor site, NPs can be internalized by tumor cells and/or release their cargo in the tumor microenvironment. It is known that the tumor microenvironment is acidic (69), and this pathological characteristic of cancer can be used as a strategy for the controlled release of NPs responsive to acidic pH (40). This strategy prevents cargo release to non-target tissues and aids in the mitigation of possible adverse effects. Kundu and co-workers (40) designed their study based on this approach by using pH-sensitive NPs. They observed that the release of curcumin from the nanohybrid zinc oxide NPs was improved in decreased pH and resulted in an increased accumulation of curcumin in tumor tissue and a significative tumor volume reduction (~77%). In addition, no biochemical modifications or structural damage were observed in the liver and kidneys (40). Huang and co-workers (48) encapsulated curcumin in pH-sensitive polymeric NPs and showed a significant reduction in tumor volume followed by increased survival time (**Table 1**) (48). Internalization of NPs can be mediated or not by active targeting ligands (depending on the mechanism triggered), and it occurs mainly through endocytosis pathways where the main mechanisms comprise a) clathrin-mediated endocytosis; b) caveolae-mediated endocytosis, for NPs up to 200 nm; c) macropinocytosis; and d) other clathrin and caveola-independent endocytosis for NPs with sizes between 250 nm and 3 µm (70, 71). Once inside the cells, NPs can interact with specific organelles and/or release their cargo to reach potential targets, such as the ones involved in cell death/survival and cell proliferation pathways (66).

Curcumin can modulate multiple apoptosis signaling pathways. The predominant apoptotic mechanism—extrinsic (receptor-mediated) or intrinsic (mitochondrial) – differs between cell types, differentiation stages, or curcumin concentrations. Increase of Bax/Bcl-2 ratio, activation of caspase-3, inhibition of telomerase, DNA fragmentation, and induction of redox signaling are some of the apoptotic mechanisms activated by curcumin in distinct breast cancer cells (72–74). Cell cycle arrest by free curcumin has also been described and is potentially associated with its antiproliferative effects (74). Regarding the antitumor mechanisms reported in the included studies, Cur-NPs were shown to induce at least tumor apoptosis, necrosis, and/or cell proliferation blockage in *in vivo* breast cancer models (26, 28, 30, 34, 36, 37, 40, 41, 45, 48).

Angiogenesis involves the development of new blood vessels from pre-existing vessels and plays an important role in tumor growth, maintenance, and metastasis (26, 30). Free curcumin has been described presenting anti-angiogenesis effects by inhibiting or modulating many pro-angiogenesis factors such as vascular endothelial growth factor (VEGF), matrix metalloproteinases (MMPs), and basic fibroblast growth factor (bFGF) in *in vitro* and *in vivo* studies (26, 30, 58). Similar effects were reported in the included studies when curcumin was associated with micelles, graphene oxide, or polymeric NPs and administered in *in vivo* breast cancer models (28, 34, 36, 41, 45).

It is known that a population of cancer stem cells (CSCs) is present within the tumor microenvironment. These cells are able to activate self-sustaining and self-renewal mechanisms, giving rise to heterogeneous cancer cells that comprise the tumor (75). CSCs are also known to present a high expression of P-glycoprotein, a well-known protein involved in multidrug resistance (MDR), making them less susceptible to antitumor therapies (76). Interestingly, free curcumin has been described affecting CSCs with no toxicity to normal stem cells. The mechanisms involve modulation of P-glycoprotein (77); suppression of the release of cytokines such as interleukin (IL)-6, IL-8, and IL-1, which stimulate CSCs; among others (75). Cur-NPs of the studies evaluated herein seems to maintain this property since studies with curcumin associated with micelles and polymeric NPs have shown a significant reduction in the proportion of CSCs present in *in vivo* breast cancer models (26, 33) (**Table 1**).

Metastasis is the process where cells from the primary tumor spread to distant sites and give rise to a secondary tumor. Advanced breast cancer includes both stage (IV) of metastatic breast cancer and inoperable locally advanced breast cancer, which has not spread to distant organs. The most common site affected by breast cancer cells are the axillary lymph nodes, lungs, liver, and bones (1, 78). In the present review, Cur-NPs showed significant effects against tumor metastasis in *in vivo* breast cancer models. For instance, curcumin associated with graphene oxide NPs reduced the regions of metastasis in a triple negative breast cancer model (34).

Different routes of administration were adopted among the studies evaluated herein. The oral administration route is preferred over other routes for drugs and NPs due to advantages such as ease of ingestion, good patient compliance, and pain avoidance. The main limitation of this route is the knowledge of the real dose absorbed (79). Only one included study explored oral administration with curcumin entrapped in a lipid-based NP (25). Interestingly, a remarkable tumor volume reduction of ~60% was achieved; nevertheless, the dose of curcumin administered was the highest among all the other studies (100 mg/Kg), probably due to variations in NP absorption through this route.

Other routes of administration can be applied to avoid the gastrointestinal tract and potential degradation (80). Intratumoral administration is an interesting option for breast cancer therapy. NPs can be administered with a minimal invasive procedure with a regular biopsy needle, for example, right in the tumor site, increasing the lifetime of drugs in contact with malignant target cells, reducing adverse effects on healthy tissues, and bypassing liver metabolism (81). Two of the included studies used this route to administer micelle NPs to triple negative breast cancer models and showed similar outcomes in terms of tumor volume reduction (51–60%) (26, 27).

The intraperitoneal (IP) route is commonly used in rodents and consists of an injection of pharmacological drugs into the peritoneal cavity. Quick and minimally stressful for animals, the IP route permits safe administration of a large volume of drug and it is more appropriate when the intravenous route is challenging. The IP route is an entry portal for blood circulation through the capillary system (80). Two of the included studies reported the success of Cur-micelle NP administration *via* IP route with a reduction of tumor volume of ~80 and ~59.1%, respectively (28, 29).

The intravenous (IV) route of administration enables the rapid circulation of an administered drug in the bloodstream. Although approximately 70% of the included studies adopted this route of administration, precise efficacy comparisons regarding composition and other characteristics (*e.g.*, HD, PDI, PZ) of NPs are limited due to variations in cell lines, number of cells used for induction, moment of the first treatment, and dose/treatment regimens. However, some comparisons involving dose concentrations and presence of active targeting were possible when analyzing studies that evaluated more than one experimental variable in the same experimental design. In general, it was observed that all the types of NPs used led to improved outcomes in terms of tumor volume reduction in models of estrogen receptor (ER) positive, chemically induced, and triple negative breast cancer (**Table 1**).

MCF-7 is one of the human cell lines most commonly used for breast cancer research, since it expresses substantial levels of estrogen receptor (ER) mimicking the majority of breast cancers diagnosed nowadays (82). Analyzing MCF-7 models of the included studies, it was observed that the influence of active targeting in improved efficacy outcomes depends on the moiety used. Lin and co-workers (50) reported significant improvements in tumor volume reduction when attaching FA, as the targeting moiety, to lipid-based NPs (~83%) when compared to non-targeted NPs (~66%) (50). Similar results were observed in MDA-MB-231 *in vivo* models (triple negative breast cancer), where the presence of folate, as an active targeting moiety, showed improved tumor volume reduction (~90%) when compared to non-targeted NPs (~75%) (46). On the other hand, no significant improvements were observed when peptide moieties with affinity to EGFR were used in polymeric NPs when compared with non-targeted NPs (37). Another interesting aspect that seems to improve efficacy outcomes in MCF-7 models is the design of pH-sensitive NPs. Yu and co-workers (47) reported improved reduction of tumor volume in animals treated with pH-sensitive micelles (mPEG-PLA with PAE) (~65.6%) when compared to the ones treated with non-pH-sensitive micelles (~47.1%) (47).

Both MCF-7 and MDA-MB-231 conventional *in vivo* models are induced in immunocompromised mice due to the human origin of these cell lines (83). These models, also known as xenograft, lack relevance when the study aims to evaluate/associate the outcomes with a functional immune system. In this case, syngeneic models, where cells of the same genetic background (murine) are implanted into a mouse with a native immune system, are recommended (83). Syngeneic breast cancer models usually use the 4T1 cell line as a representative model that mimics triple negative breast cancers (84). Analyzing 4T1 models used in the included studies, it was observed that the outcome of tumor volume reduction showed a tendency to respond in a dose-dependent manner. Greish and co-workers (44) showed that a 20mg/Kg dose led to improved tumor volume reduction (~92%) when compared to a 10 mg/ml dose (~61%) (44). Nevertheless, when comparing high doses, such as 40 and 80 mg/Kg, no significant improvements were observed between them (49). The presence of the active targeting moieties FA or HA in the NPs showed improved efficacy, as reported by Laha and co-workers (31) and Ji and co-workers (31, 35).

Analyzing only the studies of Sahne and co-workers (34), Ji and co-workers (35) and He and co-workers (36), all with breast cancer induction with 4T1 cells (10^6^) in Balb/c mice, and with the treatments performed intravenously and in very similar doses, 4 or 5 mg/kg, but with a difference in the treatment schedule (**Table 1**), (in the work of Sahne and co-workers, the treatment was daily, for 21 days; in the work of Ji and co-workers, the treatment was every two days for 10 days; and in the work of He and co-workers, it was every four days in 21 total days), it can be observed that, interestingly, the treatments with the Cur-NPs, either by FA-GO-NP or HA-Cur-NP, promoted a similar percentage of reduction in tumor volume (approximately 86%, **Table 1**). Assessing NPs, HA-Cur-NP is a nanocrystal that has a 162 nm HD, while FA-GO-NP is a graphene oxide NP that has a 60 nm HD. In the treatment aspect, treatment every 2 days for only 10 days of HA-Cur-NP had the same antitumor efficiency as daily treatment for 21 days of FA-GO-NP. Therefore, it is a shorter and less aggressive therapeutic regimen, presenting similar efficiency (34–36).

The elimination of NPs occurs in organs and tissue systems after i.v. injection by two main clearance systems: reticuloendothelial system (RES) or mononuclear phagocyte system (MPS) and by renal and hepatic systems. Properties of NPs, including core type, surface chemistry, size, shape, degradability, and surface charge influence the process of clearance (85). The MPS is based on phagocytosis (mostly for NPs between 50 and 200 nm) or pinocytosis, and degraded NPs are excreted into the blood circulation, decreasing the injected dose (85, 86). Renal and hepatic systems are the main clearance organs of NPs less than 100 nm through glomerular filtration and tubular secretion in the kidney. NPs that are not cleared by the kidney can be processed in the liver due to the presence of a large number of Kupffer cells that can sequester foreign bodies, and the very permeable sinusoidal endothelial cells that enhance liver uptake and retention of NPs (85).

Among the included studies that evaluated toxicity effects, none of the Cur-NPs provoked toxicity, considering biochemical markers, hematological changes, damage to major organs, and weight loss (**Table 1**). It is worth pointing out that the majority of studies (~80%) analyzing the efficacy of Cur-NPs also evaluated at least one toxicity outcome, showing that research into NPs is not only interested in treatment efficacy but also considers safety issues. Nevertheless, ~33% of such studies used only body weight as a parameter for toxicity analysis. Thus, it is important to pursue deeper investigations beyond these parameters in order to understand better the safety of the treatment and enable its clinical translation.

## Limitations

Some limitations were encountered during the elaboration of this systematic review. First, there was high heterogeneity regarding NP type, characteristics of NPs, animal models, period of administration, and intervention concentrations which made meta‐analysis unfeasible. Furthermore, one study was excluded in phase 2 because its full copy could not be obtained. Moreover, most SYRCLE’ RoB criteria were unclearly reported in most included studies therefore, limiting the evaluation of study quality. 

## Conclusion

This systematic review evidences that the use of NPs as drug delivery systems for curcumin is a promising approach for the treatment of breast cancer. The results show significant tumor volume reduction in all breast cancer models, which could be attributed to increased apoptosis and necrosis rate, reduction of tumor cell proliferation and impairment of angiogenesis, and even reduction of the population of stem cancer cells, which might also be correlated with improved survival times. All of these improved outcomes are also related to no or low adverse effects in terms of body weight, histopathology of major organs (*e.g.*, liver, kidneys, lungs, spleen), or alterations in hematological/biochemical parameters.

Variations in NP structure should be considered according to the type of breast tumor as well as the route of administration and dose schedule. In addition, the cost-effective and large-scale manufacturing of the proposed NP platforms is also of considerable importance to enable a real translation of these remarkable technologies from the bench to the bedside.

Although Cur-NPs’ association with other therapeutic approaches is not within the scope of the present work, it is recommended that systematic evaluations of outcomes regarding efficacy and toxicity of Cur-NPs when associated with other plant-derived molecules or currently prescribed therapies (*e.g.*, chemotherapy, radiotherapy) should be further considered. Altogether, this systematic review supports the proposal that Cur-NPs provide an effective and safe therapeutic approach in *in vivo* models of breast cancer, reinforcing the currently available evidence that their usage should be further analyzed in clinical trials for breast cancer treatments.

## Data Availability Statement

The original contributions presented in the study are included in the article/**Supplementary Material**. Further inquiries can be directed to the corresponding author.

## Author Contributions

AO, VS, GL, and GJ conceived the idea and prepared, edited, and finalized the manuscript. The articles were selected in two phases: screening of titles and abstracts (phase 1), and full-text reading (phase 2). In phase 1, two authors (AO and VS) reviewed titles and abstracts of all references identified in the electronic databases and selected articles that seemed to meet the inclusion criteria. In phase 2, six pairs of authors (AO and VS; LA and GF; WP and AP; JO and MG; MS and GG; PC and GJ) were formed to independently analyze the full text of articles selected in phase 1 and exclude studies that did not meet the inclusion criteria. LA and PC revised the manuscript. GG prepared the graphical abstract. All authors contributed to the article and approved the submitted version.

## Funding

This work was supported by Coordenação de Aperfeiçoamento de Pessoal de Nível Superior (CAPES—Finance Code 001), Conselho Nacional de Desenvolvimento Cientifico e Tecnológico (CNPq), Instituto Nacional de Ciência e Tecnologia em Nanobiotecnologia (INCT Nanobiotecnologia), and Fundação de Amparo à Pesquisa do Distrito Federal (FAP-DF). The funders had no role in study design, data collection and analysis, decision to publish, or preparation of the manuscript.

## Conflict of Interest

The authors declare that the research was conducted in the absence of any commercial or financial relationships that could be construed as a potential conflict of interest.
