# Supplementary material for: In Vivo Efficacy and Toxicity of Curcumin Nanoparticles in Breast Cancer Treatment: A Systematic Review
Source: Front Oncol. 2021 Mar 9;11:612903. doi: 10.3389/fonc.2021.612903 (PMC7986721; doi:10.3389/fonc.2021.612903)
Supplement: Supplementary file 1 [file Table_1.docx]

**Table S1**. Database Search and results.

| **Database** | **Search** | **Quantity of publications** |
| --- | --- | --- |
| **PUBMED**    **(August 10, 2020**  **Update:)** | ("Breast Cancer" OR "Breast Neoplasm" OR “Mammary Cancer” OR “Malignant Neoplasm of Breast” OR (“Mammary Carcinoma” AND Human) OR “Breast Carcinoma” OR “Cancer of Breast”) AND (Curcumin OR “Turmeric Yellow” OR (Yellow AND Turmeric) OR Diferuloylmethane) AND (Nanoparticles OR Nanoparticle OR Nanogels OR "Nanocomposite Gels" OR "Nanocomposite Gel" OR Nanocapsule OR Nanocapsules OR Nanoemulsion OR Micelle OR Micelles OR Liposome OR Liposomal) AND "in vivo" | 55 |
| **LILACS**  **(August 10, 2020**  **Update: August 14, 2020)** | (tw:(Câncer de mama)) AND (tw:(Nano*)) AND (tw:(Curcumina)) OR (tw:(Turmeric)) AND (tw:(In vivo)) | 35 |
| **MEDLINE**  **EMBASE**  **SCOPUS**  **PROQUEST**  **CINAHL**  **FSTA**    **(August 11, 2020**  **Update: August 14, 2020)** | ("Breast Cancer" OR "Breast Neoplasm" OR “Mammary Cancer” OR “Malignant Neoplasm of Breast” OR (“Mammary Carcinoma” AND Human) OR “Breast Carcinoma” OR “Cancer of Breast”) AND (Curcumin OR “Turmeric Yellow” OR (Yellow AND Turmeric) OR Diferuloylmethane) AND (Nanoparticles OR Nanoparticle OR Nanogels OR "Nanocomposite Gels" OR "Nanocomposite Gel" OR Nanocapsule OR Nanocapsules OR Nanoemulsion OR Micelle OR Micelles OR Liposome OR Liposomal) AND "in vivo" | 52 |
|  |  | 113 |
|  |  | 112 |
|  |  | 1 |
|  |  | 1 |
|  |  | 1 |
| **SCIENCE DIRECT**    **(August 10, 2020**  **Update: August 10, 2020)** | ("breast cancer" OR "breast neoplasm" OR  "cancer of breast) AND (curcumin OR turmeric) AND (nano OR micelle OR nanocapsule OR nanoparticle) | 57 |
| **WEB OF SCIENCE**  **(August 10, 2020**  **Update: August 14, 2020)** | ("Breast Cancer" OR "Breast Neoplasm") AND (Curcumin OR “Turmeric Yellow” OR (Yellow AND Turmeric) OR Diferuloylmethane) AND (Nanoparticles OR Nanoparticle OR Nanogels OR "Nanocomposite Gels" OR "Nanocomposite Gel" OR Nanocapsule OR Nanocapsules OR Nanoemulsion OR Micelle OR Micelles OR Liposome OR Liposomal) AND "in vivo" | 41 |
| **PORTAL REGIONAL DA BSV**  **(August 10, 2020**  **Update: August 14, 2020)** | ("Breast Cancer" OR "Breast Neoplasm") AND (Curcumin OR “Turmeric Yellow” OR (Yellow AND Turmeric) OR Diferuloylmethane) AND (Nanoparticles OR Nanoparticle OR Nanogels OR "Nanocomposite Gels" OR "Nanocomposite Gel" OR Nanocapsule OR Nanocapsules OR Nanoemulsion OR Micelle OR Micelles OR Liposome OR Liposomal) AND "in vivo" | 60 |
