# Supplementary material for: In Vivo Efficacy and Toxicity of Curcumin Nanoparticles in Breast Cancer Treatment: A Systematic Review
Source: Front Oncol. 2021 Mar 9;11:612903. doi: 10.3389/fonc.2021.612903 (PMC7986721; doi:10.3389/fonc.2021.612903)
Supplement: Supplementary file 2 [file Table_2.docx]

**Table S2.** Excluded articles and reasons for exclusion (n=50).

| Authors | Reason for exclusion |
| --- | --- |
| 1. Abdel-Hafez, Salma M. et al., 2018 (1) | 6 |
| 1. Amani, Soheil et al., 2019 (2) | 6 |
| 1. Chen, Sunhui et al., 2017 (3) | 3 |
| 1. Chidambaram, Moorthi et al., 2014 (4) | 3 |
| 1. Dhule, Santosh S. et al., 2012 (5) | 1 |
| 1. Ding, Lingling et al., 2016 (6) | 3 |
| 1. Duan, Dongyu et al., 2018 (7) | 4 |
| 1. Dutta, Bijaideep et al., 2020 (8) | 6 |
| 1. Galisteo-González, F. et al., 2018 (9) | 3 |
| 1. Gao, Cheng et al., 2017 (10) | 4 |
| 1. Gao, Jiafeng et al., 2019 (11) | 3 |
| 1. Gao, Xiao Ping et al., 2014 (12) | 2 |
| 1. Guo, Fangyuan et al., 2019 (13) | 4 |
| 1. Guo, Qingfa et al., 2014 (14) | 3 |
| 1. Guo, Shengrong et al., 2015 (15) | 3 |
| 1. Gupta, Vishal et al., 2009 (16) | 6 |
| 1. Hossain et al., 2015 (17) | 4 |
| 1. Jithan, Av et al., 2011 (18) | 6 |
| 1. Kamalabadi-Farahani, Mohammad et al., 2018 (19) | 6 |
| 1. Kamble, Sonali et al., 2016 (20) | 6 |
| 1. Kendre, Prakash et al., 2015 (21) | 6 |
| 1. Kumari, Preeti et al., 2017 (22) | 1 |
| 1. Kurzrock, R. et al., 2005 (23) | 1 |
| 1. Lecot, Nicole et al., 2020 (24) | 4 |
| 1. Li, Hui et al., 2016 (25) | 3 |
| 1. Li, Lan et al., 2005 (26) | 1 |
| 1. Liu, Mengna et al., 2019 (27) | 6 |
| 1. Martey, Orleans et al., 2017 (28) | 8 |
| 1. Mengjiao, Qi et al., 2018 (29) | 7 |
| 1. Nejadshafiee, Vajihe et al., 2019 (30) | 1 |
| 1. Nguyen, Ngoc The et al., 2018 (31) | 3 |
| 1. Nosrati, Hamed et al., 2019 (32) | 3 |
| 1. Palange, Anna L. et al., 2014 (33) | 6 |
| 1. Pawar, Harish et al., 2016 (34) | 3/4 |
| 1. Rastegar, Roghayeh et al., 2018 (35) | 3 |
| 1. Rejinold et al., 2016 (36) | 4 |
| 1. Sahu et al., 2016 (37) | 9 |
| 1. Song, Zhiwang et al., 2016 (38) | 1 |
| 1. Sorasitthiyanukarn, Feuangthit Niyamissara et al., 2018 (39) | 6 |
| 1. Sun, Jiabei et al., 2013 (40) | 4 |
| 1. Sun, Madi et al., 2019 (41) | 6 |
| 1. Thadakapally, R. et al., 2016 (42) | 4 |
| 1. Thamake, S.I. Sanjay I. et al., 2012 (43) | 3 |
| 1. Vemuri, Satish Kumar et al., 2019 (44) | 6 |
| 1. Wang, Jiao et al., 2016 (45) | 4 |
| 1. Wang, Zhaoxian et al., 2018 (46) | 8 |
| 1. Yin, Hai Tao et al., 2013 (47) | 1 |
| 1. Zhang, Beibei et al., 2019 (48) | 3 |
| 1. Zhang, Jingjing et al., 2014 (49) | 3 |
| 1. Zhang, Xiwen et al., 2013 (50) | 8 |

Legend: 1- Breast cancer was not used; 2- Only free curcumin was used, and not nanoencapsulated curcumin; 3- Nanocurcumin was used with other anticancer substances, molecules, or therapy; 4- Nanocurcumin was not evaluated for treatment efficacy and/or toxicity effect; 5- Reviews, letters, personal opinions, book chapters, conference abstracts, and patents; 6- Only *in vitro* study or Clinical trials; 7- Full copy was not available; 8- Used of analog of curcumin; 9- Low quality.

References (Table S2)

1. Abdel-Hafez SM, Hathout RM, Sammour OA. Curcumin-loaded ultradeformable nanovesicles as a potential delivery system for breast cancer therapy. Colloids Surfaces B Biointerfaces. 2018 Jul 1;167:63–72.

2. Amani S, Mohamadnia Z, Mahdavi A. pH-responsive hybrid magnetic polyelectrolyte complex based on alginate/BSA as efficient nanocarrier for curcumin encapsulation and delivery. Int J Biol Macromol. 2019 Dec 1;141:1258–70.

3. Chen S, Liang Q, Liu E, Yu Z, Sun L, Ye J, et al. Curcumin/sunitinib co-loaded BSA-stabilized SPIOs for synergistic combination therapy for breast cancer. J Mater Chem B. 2017;5(22):4060–72.

4. Chidambaram M, Krishnasamy K. Codelivery of nanosized curcumin and bioenhancer using acid degradable polymeric nanoparticles displayed enhanced anticancer efficacy. Nano Biomed Eng. 2014;6(2):1–13.

5. Dhule SS, Penfornis P, Frazier T, Walker R, Feldman J, Tan G, et al. Curcumin-loaded γ-cyclodextrin liposomal nanoparticles as delivery vehicles for osteosarcoma. 2012 May [cited 2020 Aug 14];8(4). Available from: http://www.ncbi.nlm.nih.gov/pubmed/21839055

6. Ding L, Li J, Huang R, Liu Z, Li C, Yao S, et al. Salvianolic acid B protects against myocardial damage caused by nanocarrier TiO2; and synergistic anti-breast carcinoma effect with curcumin via codelivery system of folic acid-targeted and polyethylene glycol-modified TiO2 nanoparticles. Int J Nanomedicine. 2016 Nov 2;11:5709–27.

7. Duan D, Wang A, Ni L, Zhang L, Yan X, Jiang Y, et al. Trastuzumab- and fab’ fragment-modified curcumin PEG -PLGA nanoparticles: Preparation and evaluation in vitro and in vivo. Int J Nanomedicine [Internet]. 2018 Mar 22 [cited 2020 Aug 14];13:1831–40. Available from: https://pubmed.ncbi.nlm.nih.gov/29606874/

8. Dutta B, Rawoot YA, Checker S, Shelar SB, Barick KC, Kumar S, et al. Micellar assisted aqueous stabilization of iron oxide nanoparticles for curcumin encapsulation and hyperthermia application. Nano-Structures and Nano-Objects. 2020 Apr 1;22.

9. Galisteo-González F, Molina-Bolívar JA, Navarro SA, Boulaiz H, Aguilera-Garrido A, Ramírez A, et al. Albumin-covered lipid nanocapsules exhibit enhanced uptake performance by breast-tumor cells. Colloids Surfaces B Biointerfaces. 2018 May 1;165:103–10.

10. Gao C, Tang F, Gong G, Zhang J, Hoi MPM, Lee SMY, et al. PH-Responsive prodrug nanoparticles based on a sodium alginate derivative for selective co-release of doxorubicin and curcumin into tumor cells. Nanoscale. 2017 Sep 14;9(34):12533–42.

11. Gao J, Fan K, Jin Y, Zhao L, Wang Q, Tang Y, et al. PEGylated lipid bilayer coated mesoporous silica nanoparticles co-delivery of paclitaxel and curcumin leads to increased tumor site drug accumulation and reduced tumor burden. Eur J Pharm Sci. 2019 Dec 1;140.

12. Gao XP, Feng F, Zhang XQ, Liu XX, Wang Y Bin, She JX, et al. Toxicity assessment of 7 anticancer compounds in zebrafish. Int J Toxicol [Internet]. 2014 [cited 2020 Aug 15];33(2):98–105. Available from: https://pubmed.ncbi.nlm.nih.gov/24563414/

13. Guo F, Fu Q, Jin C, Ji X, Yan Q, Yang Q, et al. Dual functional matrix metalloproteinase-responsive curcumin-loaded nanoparticles for tumor-targeted treatment. Drug Deliv. 2019 Jan 1;26(1):1027–38.

14. Guo Q, Li X, Yang Y, Wei J, Zhao Q, Luo F, et al. Enhanced 4T1 breast carcinoma anticancer activity by co-delivery of doxorubicin and curcumin with core-shell drug-carrier based on heparin modified poly(l-lactide) grafted polyethylenimine cationic nanoparticles. J Biomed Nanotechnol. 2014;10(2):227–37.

15. Guo S, Lv L, Shen Y, Hu Z, He Q, Chen X. A nanoparticulate pre-chemosensitizer for efficacious chemotherapy of multidrug resistant breast cancer. Sci Rep. 2016;6(September 2015):1–11.

16. Gupta V, Aseh A, Ríos CN, Aggarwal BB, Mathur AB. Fabrication and characterization of silk fibroin-derived curcumin nanoparticles for cancer therapy. 2009;4.

17. Hossain DMS, Panda AK, Chakrabarty S, Bhattacharjee P, Kajal K, Mohanty S, et al. MEK inhibition prevents tumour-shed transforming growth factor-β-induced T-regulatory cell augmentation in tumour milieu. Immunology. 2015 Apr 1;144(4):561–73.

18. Jithan A, Madhavi K, Madhavi M, Prabhakar K. Preparation and characterization of albumin nanoparticles encapsulating curcumin intended for the treatment of breast cancer. 2011 Apr [cited 2020 Aug 17];1(2). Available from: http://www.ncbi.nlm.nih.gov/pubmed/23071931

19. Kamalabadi-Farahani M, Vasei M, Ahmadbeigi N, Ebrahimi-Barough S, Soleimani M, Roozafzoon R. Anti-tumour effects of TRAIL-expressing human placental derived mesenchymal stem cells with curcumin-loaded chitosan nanoparticles in a mice model of triple negative breast cancer. Artif Cells, Nanomedicine Biotechnol [Internet]. 2018;46(sup3):S1011–21. Available from: https://doi.org/10.1080/21691401.2018.1527345

20. Kamble S, Utage B, Mogle P, Kamble R, Hese S, Dawane B, et al. Evaluation of Curcumin Capped Copper Nanoparticles as Possible Inhibitors of Human Breast Cancer Cells and Angiogenesis: a Comparative Study with Native Curcumin. AAPS PharmSciTech [Internet]. 2016 Oct 1 [cited 2020 Aug 14];17(5). Available from: http://www.ncbi.nlm.nih.gov/pubmed/26729534

21. Kendre P, D CP. Emerging trend in adressing the challenges to oral nanocurcumin delivery to improve quality of life of patients suffering from cancer Enhancement of drug solubility View project EMERGING TREND IN ADRESSING THE CHALLENGES TO ORAL NANOCURCUMIN DELIVERY TO I [Internet]. Available from: https://www.researchgate.net/publication/305147687

22. Kumari P, Muddineti OS, Rompicharla SVK, Ghanta P, Adithya KBBN, Ghosh B, et al. Cholesterol-conjugated poly(D, L-lactide)-based micelles as a nanocarrier system for effective delivery of curcumin in cancer therapy. Drug Deliv [Internet]. 2017 Feb 3 [cited 2020 Aug 14];24(1):209–23. Available from: https://pubmed.ncbi.nlm.nih.gov/28156164/

23. Kurzrock R, Li L. Liposome-encapsulated curcumin: in vitro and in vivo effects on proliferation, apoptosis, signaling, and angiogenesis. J Clin Oncol. 2005 Jun;23(16_suppl):4091–4091.

24. Lecot N, Glisoni R, Oddone N, Benech J, Fernández M, Gambini JP, et al. Glucosylated Polymeric Micelles Actively Target a Breast Cancer Model. Adv Ther. 2020 May 13;2000010.

25. Li H, Tian J, Wu A, Wang J, Ge C, Sun Z. Self-assembled silk fibroin nanoparticles loaded with binary drugs in the treatment of breast carcinoma. Int J Nanomedicine. 2016 Sep 2;11:4373–80.

26. Li L, Braiteh FS, Kurzrock R. Liposome-encapsulated curcumin: In vitro and in vivo effects on proliferation, apoptosis, signaling, and angiogenesis. Cancer. 2005;104(6):1322–31.

27. Liu M, Wang B, Guo C, Hou X, Cheng Z, Chen D. Novel multifunctional triple folic acid, biotin and CD44 targeting pH-sensitive nano-actiniaes for breast cancer combinational therapy. Drug Deliv [Internet]. 2019;26(1):1002–16. Available from: https://doi.org/10.1080/10717544.2019.1669734

28. Martey O, Nimick M, Taurin S, Sundararajan V, Greish K, Rrosengren RJ. Styrene maleic acid-encapsulated RL71 micelles suppress tumor growth in a murine xenograft model of triple negative breast cancer. Int J Nanomedicine. 2017 Oct 4;12:7225–37.

29. Qi M, Zou S, Guo C, Wang K, Yu Y, Zhao F, et al. Enhanced in vitro and in vivo anticancer properties by using a nanocarrier for co-delivery of antitumor polypeptide and curcumin. J Biomed Nanotechnol. 2018 Jan 1;14(1):139–49.

30. Nejadshafiee V, Naeimi H, Goliaei B, Bigdeli B, Sadighi A, Dehghani S, et al. Magnetic bio-metal–organic framework nanocomposites decorated with folic acid conjugated chitosan as a promising biocompatible targeted theranostic system for cancer treatment. Mater Sci Eng C [Internet]. 2019 Jun 1 [cited 2020 Aug 14];99:805–15. Available from: https://linkinghub.elsevier.com/retrieve/pii/S0928493118323336

31. Nguyen NT, Nguyen NNT, Tran NTN, Le PN, Nguyen TBT, Nguyen NH, et al. Synergic activity against MCF-7 breast cancer cell growth of nanocurcumin-encapsulated and cisplatin-complexed nanogels. Molecules. 2018 Dec 18;23(12).

32. Nosrati H, Abhari F, Charmi J, Davaran S, Danafar H. Multifunctional nanoparticles from albumin for stimuli-responsive efficient dual drug delivery. Bioorg Chem. 2019 Jul 1;88.

33. Palange AL, Di Mascolo D, Carallo C, Gnasso A, Decuzzi P. Lipid-polymer nanoparticles encapsulating curcumin for modulating the vascular deposition of breast cancer cells. Nanomedicine Nanotechnology, Biol Med. 2014;10(5):e991–1002.

34. Pawar H, Surapaneni SK, Tikoo K, Singh C, Burman R, Gill MS, et al. Folic acid functionalized long-circulating co-encapsulated docetaxel and curcumin solid lipid nanoparticles: In vitro evaluation, pharmacokinetic and biodistribution in rats. Drug Deliv. 2016 May 3;23(4):1453–68.

35. Rastegar R, Akbari Javar H, Khoobi M, Dehghan Kelishadi P, Hossein Yousefi G, Doosti M, et al. Evaluation of a novel biocompatible magnetic nanomedicine based on beta-cyclodextrin, loaded doxorubicin-curcumin for overcoming chemoresistance in breast cancer. Artif Cells, Nanomedicine Biotechnol [Internet]. 2018;46(sup2):207–16. Available from: https://doi.org/10.1080/21691401.2018.1453829

36. Rejinold NS, Thomas RG, Muthiah M, Lee HJ, Jeong YY, Park I, et al. Breast Tumor Targetable Fe3O4 Embedded Thermo-Responsive Nanoparticles for Radiofrequency Assisted Drug Delivery. 2016 Jan 1 [cited 2020 Aug 14];12(1):43–55. Available from: http://www.ncbi.nlm.nih.gov/pubmed/27301171

37. Sahu BP, Hazarika H, Bharadwaj R, Loying P, Baishya R, Dash S, et al. Curcumin-docetaxel co-loaded nanosuspension for enhanced anti-breast cancer activity. Expert Opin Drug Deliv. 2016 Aug 2;13(8):1065–74.

38. Song Z, Lu Y, Zhang X, Wang H, Han J, Dong C. Novel curcumin-loaded human serum albumin nanoparticles surface functionalized with folate: Characterization and in vitro/vivo evaluation. Drug Des Devel Ther. 2016 Aug 17;10:2643–9.

39. Sorasitthiyanukarn FN, Muangnoi C, Ratnatilaka Na Bhuket P, Rojsitthisak PP, Rojsitthisak PP. Chitosan/alginate nanoparticles as a promising approach for oral delivery of curcumin diglutaric acid for cancer treatment. Mater Sci Eng C. 2018 Dec 1;93:178–90.

40. Sun J, Bi C, Chan HM, Sun S, Zhang Q, Zheng Y. Curcumin-loaded solid lipid nanoparticles have prolonged in vitro antitumour activity, cellular uptake and improved in vivo bioavailability. Colloids Surfaces B Biointerfaces. 2013 Nov 1;111:367–75.

41. Sun M, Zhang Y, He Y, Xiong M, Huang H, Pei S, et al. Green synthesis of carrier-free curcumin nanodrugs for light-activated breast cancer photodynamic therapy. Colloids Surfaces B Biointerfaces. 2019 Aug 1;180:313–8.

42. Thadakapally R, Aafreen A, Aukunuru J, Habibuddin M, Jogala S. Preparation and Characterization of PEG-albumin-curcumin Nanoparticles Intended to Treat Breast Cancer. 2016 Jan 1 [cited 2020 Aug 14];78(1):65–72. Available from: http://www.ncbi.nlm.nih.gov/pubmed/27168683

43. Thamake SISI, Raut SLSL, Gryczynski Z, Ranjan APAP, Vishwanatha JKJK. Alendronate coated poly-lactic-co-glycolic acid (PLGA) nanoparticles for active targeting of metastatic breast cancer. Biomaterials [Internet]. 2012 Oct [cited 2020 Aug 14];33(29):7164–73. Available from: https://pubmed.ncbi.nlm.nih.gov/22795543/

44. Vemuri SK, Banala RR, Mukherjee S, Uppula P, GPV S, Gurava GR, et al. Novel biosynthesized gold nanoparticles as anti-cancer agents against breast cancer: Synthesis, biological evaluation, molecular modelling studies. Mater Sci Eng C. 2019 Jun 1;99:417–29.

45. Wang J, Wang Y, Liu Q, Yang L, Zhu R, Yu C, et al. Rational Design of Multifunctional Dendritic Mesoporous Silica Nanoparticles to Load Curcumin and Enhance Efficacy for Breast Cancer Therapy. 2016 Oct 12 [cited 2020 Aug 14];8(40):26511–23. Available from: http://www.ncbi.nlm.nih.gov/pubmed/27619078

46. Wang Z, Sau S, Alsaab HO, Iyer AK. CD44 directed nanomicellar payload delivery platform for selective anticancer effect and tumor specific imaging of triple negative breast cancer. Nanomedicine Nanotechnology, Biol Med. 2018 Jun 1;14(4):1441–54.

47. Yin HT, Zhang D geng, Wu X li, Huang XE, Chen G. In vivo evaluation of curcumin-loaded nanoparticles in a A549 xenograft mice model. Asian Pacific J Cancer Prev [Internet]. 2013 [cited 2020 Aug 15];14(1):409–12. Available from: https://pubmed.ncbi.nlm.nih.gov/23534763/

48. Zhang B, Xu C, Sun C, Yu C. Polyphosphoester-Based Nanocarrier for Combined Radio-Photothermal Therapy of Breast Cancer. ACS Biomater Sci Eng. 2019 Apr 8;5(4):1868–77.

49. Zhang J, Yang Y, Fu H. CIK cell therapy for solid tumor. Vol. 6, Nano Biomedicine and Engineering. Open Access House of Science and Technology; 2014. p. 60–6.

50. Zhang X, Tian W, Cai X, Wang X, Dang W, Tang H, et al. Hydrazinocurcumin Encapsuled Nanoparticles “Re-Educate” Tumor-Associated Macrophages and Exhibit Anti-Tumor Effects on Breast Cancer Following STAT3 Suppression. PLoS One [Internet]. 2013 Jun 25 [cited 2020 Aug 14];8(6):65896. Available from: /pmc/articles/PMC3692525/?report=abstract
