# Supplementary material for: In Vivo Efficacy and Toxicity of Curcumin Nanoparticles in Breast Cancer Treatment: A Systematic Review
Source: Front Oncol. 2021 Mar 9;11:612903. doi: 10.3389/fonc.2021.612903 (PMC7986721; doi:10.3389/fonc.2021.612903)
Supplement: Supplementary file 3 [file Table_3.docx]

**Table S3**. Risk of bias of selected individual studies.

|  | Criteria used for publication risk of bias analysis* |
| --- | --- |
| 1 | Was the allocation sequence adequately generated and applied? |
| 2 | Were the groups similar at baseline or were they adjusted for confounders in the analysis? |
| 3 | Was the allocation to the different groups adequately concealed during? |
| 4 | Were the animals randomly housed during the experiment? |
| 5 | Were the caregivers and/or investigators blinded from knowledge which intervention each animal received during the experiment? |
| 6 | Were animals selected at random for outcome assessment? |
| 7 | Was the outcome assessor blinded? |
| 8 | Were incomplete outcome data adequately addressed? |
| 9 | Are reports of the study free of selective outcome reporting? |
| 10 | Was the study apparently free of other problems that could result in high risk of bias? |

* Adapted or Fulfilled SYRCLE’s RoB criteria (21).
