# Supplementary material for: In Vivo Efficacy and Toxicity of Curcumin Nanoparticles in Breast Cancer Treatment: A Systematic Review
Source: Front Oncol. 2021 Mar 9;11:612903. doi: 10.3389/fonc.2021.612903 (PMC7986721; doi:10.3389/fonc.2021.612903)
Supplement: Supplementary file 4 [file Table_4.docx]

|  | **Criteria** |
| --- | --- |
| 1 | Did the study describe the methodology for encapsulating curcumin clearly and in detail? |
| 2 | Did the study perform a characterization test for nanoencapsulated curcumin? |
| 3 | Is there consistency between the treatment model and the tumor generated? |
| 4 | Are the animal model used and the storage conditions clear? |
| 5 | Was the type of induced breast cancer mentioned? |
| 6 | Was treatment time mentioned? |
| 7 | Was the route of administration of the nanostructures mentioned? |
| 8 | Was the dose of the nanoencapsulated curcumin used mentioned? |
| 9 | Were comparison groups used as negative controls? |
| 10 | Was free curcumin used as a control? |
| 11 | Does the study have a favorable result from the animal research ethics committee for the implementation of the methodology? |
| 12 | In vivo toxicity testing were employed? |
| 13 | Were comparison groups used as negative controls? |
| 14 | Is the description of the results clear? |
| 15 | Were coherent statistical methodologies used to assess outcomes? |

**Table S4**. Quality analysis of the articles included.

*Adapted from ARRIVE RoB Tool (22)
